# Development, testing, parameterisation, and calibration of a human PBPK model for the plasticiser, di-(2-ethylhexyl) terephthalate (DEHTP) using *in silico*, *in vitro* and human biomonitoring data

**DOI:** 10.3389/fphar.2023.1140852

**Published:** 2023-02-20

**Authors:** Kevin McNally, Craig Sams, Alex Hogg, George Loizou

**Affiliations:** Health and Safety Executive, Harpur Hill, Buxton, United Kingdom

**Keywords:** DEHTP, PBPK, *in silico*, *in vitro*, biomonitoring, bayesian, markov chai Monte Carlo methods

## Abstract

A physiologically based pharmacokinetic model for di-(2-ethylhexyl) terephthalate (DEHTP) based on a refined model for di-(2-propylheptyl) phthalate (DPHP) was developed to interpret the metabolism and biokinetics of DEHTP following a single oral dose of 50 mg to three male volunteers. *In vitro* and *in silico* methods were used to generate parameters for the model. For example, measured intrinsic hepatic clearance scaled from *in vitro* to *in vivo* and plasma unbound fraction and tissue:blood partition coefficients (PCs) were predicted algorithmically. Whereas the development and calibration of the DPHP model was based upon two data streams, blood concentrations of parent chemical and first metabolite and the urinary excretion of metabolites, the model for DEHTP was calibrated against a single data stream, the urinary excretion of metabolites. Despite the model form and structure being identical significant quantitative differences in lymphatic uptake between the models were observed. In contrast to DPHP the fraction of ingested DEHTP entering lymphatic circulation was much greater and of a similar magnitude to that entering the liver with evidence for the dual uptake mechanisms discernible in the urinary excretion data. Further, the absolute amounts absorbed by the study participants, were much higher for DEHTP relative to DPHP. The *in silico* algorithm for predicting protein binding performed poorly with an error of more than two orders of magnitude. The extent of plasma protein binding has important implications for the persistence of parent chemical in venous blood—inferences on the behaviour of this class of highly lipophilic chemicals, based on calculations of chemical properties, should be made with extreme caution*.* Attempting read across for this class of highly lipophilic chemicals should be undertaken with caution since basic adjustments to PCs and metabolism parameters would be insufficient, even when the structure of the model itself is appropriate. Therefore, validation of a model parameterized entirely with *in vitro* and *in silico* derived parameters would need to be calibrated against several human biomonitoring data streams to constitute a data rich source chemical to afford confidence for future evaluations of other similar chemicals using the read-across approach.

## Introduction

Phthalates, dialkyl-or dialkylarylesters of 1,2-benzenedicarboxylic acids, are a family of synthetic chemicals that are ubiquitous in the environment. They are divided into two main types which differ in the phthalic acid side chains used in the manufacture of plastics to create products of varying flexibilities and brittleness. High-molecular-weight phthalates (HMW) increase the flexibility and durability of soft PVC-products and low molecular-weight (LMW) phthalates used in personal care products to maintain the colour and fragrance or provide a film or gloss (Latini 2005; Frederiksen et al., 2007). Phthalates are used in a wide variety of commodities such as, adhesives, medical devices, building supplies, food packaging, toys, and personal care products, etc. (Schettler 2006; Wormuth et al., 2006; Heudorf et al., 2007; Alves et al., 2016).

A number of phthalates found in the environment such as, di (2-ethylhexyl) phthalate (DEHP) have been identified as endocrine disruptors in rodents (Foster 2006; Hannon et al., 2015), and to exhibit anti-androgenic, anti-estrogenic, anti-progestogenic properties, although the concentrations needed to induce adverse health effects are high compared to the concentrations measured in contemporary human biomonitoring studies (Boberg et al., 2011; Kay et al., 2013; Den Hond et al., 2015; Johns et al., 2015). A range of human health endpoints following prenatal, neonatal, childhood and adult exposures with at least one significant association reported for urinary metabolites of di-n-butyl phthalate (DBP), benzylbutyl phthalate (BzBP), diethyl phthlate (DEP) and di-isononyl phthalate (DiNP) and for three of the urinary metabolites of DEHP is collectively known as “phthalate syndrome”, (Foster 2006; Swan 2008). These endpoints include reduced number of motile sperms, infertility, and influence on the male phenotype. Many of the associations reported in humans, most of which have been in males, are consistent with the anti-androgenic action that has been discussed for several phthalates (Swan 2008). Consequently, DBP, BBP and DEHP, have been classified as toxic to reproduction category 1B according to the Classification, Labelling and Packaging (CLP) (Regulation (EC) No 1272/2008) and have been restricted in sensitive applications such as toys or childcare articles according to Regulation (EC) No 1907/2006, Annex XVII, 51. However, the demand worldwide for plasticized products is still strong. Consequently, this drives the search for alternative plasticizers with no labelling requirements, no use restrictions and with low toxicity (Malveda et al., 2015; Bui et al., 2016).

Di (2-ethylhexyl) terephthalate (DEHTP), CAS Registry No. 6422-86-2, is one such substitute plasticizer. DEHTP has a core structure of 1,4-benzene-dicarboxylic acid and is a structural isomer of DEHP which has a core structure of 1,2-benzene-dicarboxylic acid. Toxicological studies with DEHTP have not demonstrated any of the critical effects linked with DEHP toxicity (“phthalate syndrome”) (Topping et al., 1987; Barber and Topping 1995; Gray Jr et al., 2000). A tolerable daily intake (TDI) of 1000 μg/kg bw/day based upon a 2-year rodent combined toxicity/carcinogenicity study was derived by the European Food Safety Authority (EFSA) in 2008 (EFSA 2008). The TDI for DEHTP is a factor of 20 higher than the TDI for DEHP of 50 μg/kg bw/day (EFSA 2005) and a factor of 50 higher than the reference dose (RfD) of 20 μg/kg bw/d for DEHP (US EPA 1987).

Production volumes of DEHTP were predicted to rise from 2 000 metric tons in 2002 to 90,000 metric tons by 2018 (Lessmann et al., 2016) but have reached between 100,000 and 1,000,000 tons by the end of2022^1^. Consumer exposure is expected as it is already used in a wide range of applications from food contact materials, toys, medical devices, and floorings to cable insulations (Silva et al., 2019). Therefore, the continued measurement and evaluation of DEHTP exposure in humans is warranted.

Biological monitoring (BM) of human volunteers is the controlled and repeated measurement of a chemical, its metabolites, or biochemical markers in accessible matrices such as urine, blood and saliva, exhaled air and hair (Manno et al., 2010). BM is considered a superior method of exposure assessment to personal air or dermal deposition measurements. This is because BM measurements are a composite measure of multiple routes of exposure which results in more accurate estimates of body burden to be made, (Cocker and Jones 2017). BM can capture differences in individual behaviour such as, personal hygiene and work rate, in addition to inter-individual differences in physiology and metabolism (Cocker and Jones 2017). In addition, if either parent chemical or metabolite(s), is proportionately related to the ultimate toxic entity uncertainty in external exposure assessment due to inter- and intra-individual variability can also be reduced by using BM (Boogaard et al., 2011). The estimation of organ and tissue dose, known as ‘tissue dosimetry’, from BM measurement should further improve the correlation of exposure to health effects.

Tissue dosimetry can be estimated using physiologically based pharmacokinetic (PBPK) modelling. PBPK modelling is an effective means of simulating the factors that influence tissue dose within a biological organism and subsequently, its correlation with health effects (Andersen 1995; Clewell III and Andersen, 1996; Andersen 2003; Barton et al., 2007; Clewell et al., 2008; Loizou and Hogg 2011). PBPK models are powerful tools for the integration of *in vitro*, *in silico* and *in vivo* mechanistic, pharmacokinetic, and toxicological information. They are explicit mathematical descriptions of important anatomical, physiological, and biochemical determinants of chemical absorption, distribution, metabolism and elimination (ADME). Thus, PBPK modelling is increasingly being used in chemical risk assessment (RA) (Chiu et al., 2007; Loizou et al., 2008; WHO 2010).

The aim of this study was to develop a PBPK model for DEHTP based on the model structure for di-(2-propylheptyl) phthalate (DPHP) described previously, with a minor modification, to interpret the venous blood concentrations and urinary excretion of the metabolites in exposed people (McNally et al., 2021). We use the model to understand the metabolism and urinary excretion kinetics of DEHTP following a single oral dose of 50 mg DEHTP to three male volunteers in controlled study (Lessmann et al., 2016). Model parameters were calculated using *in vitro* and *in silico* methods such as, measured intrinsic hepatic clearance scaled from *in vitro* to *in vivo* and algorithmically predicted octanol–water PC (Log P_ow_) values which, in turn, were used to predict parameters such as plasma unbound fraction and tissue:blood PCs (PCs). Global sensitivity analysis (GSA) was used to assess the capability and relevance of PBPK model structure and the sensitivity of model output to *in vitro* and *in silico* derived model parameters. The outputs from this study can also contribute to the ongoing development of a good modelling practice and regulatory acceptance of PBPK in chemical safety assessment (Barton et al., 2007; Loizou et al., 2008; Barton et al., 2009; WHO 2010; Paini et al., 2017; Ellison 2018; Fabian et al., 2019; OECD 2021).

## Materials and methods

### Experimental

#### Chemicals

Human microsomes were purchased from Tebu-bio^2^ (Peterborough, UK). The microsomes were prepared from a pool of 50, mixed gender (20 mg protein ml^⁻1^) liver samples. DEHTP and MEHTP (purity 98.6%) were provided by BASF SE. All chemicals used were of analytical grade or higher; B-nicotinamide adenine dinucleotide phosphate (NADP), purity 97%, Glucose-6-phosphate, 98%–100%, Magnesium chloride, ACS reagent >99%, and Glucose-6-phosphate dehydrogenase (type V from baker’s yeast) were obtained from Sigma Aldrich. Potassium dihydrogen phosphate, analytical grade, and Di-potassium hydrogen phosphate, analytical grade, were obtained from Fisher Scientific.

#### Analysis

Samples were analysed by liquid chromatography (Shimadzu Prominence) with tandem mass spectrometry detection (AB Sciex API 3200) using electrospray ionisation. Ion optics, temperatures and gas flows were optimised on our individual system. All analyses used a Synergi Hydro-RP column dimensions => (150 × 2mm; 4 µ; Phenomenex) in conjunction with a methanol:20 mM ammonium acetate (0.1% acetic acid) gradient. Sample injection volume was 2 µl.

#### Determination of in vitro and in vivo intrinsic clearance

**FIGURE 1:**
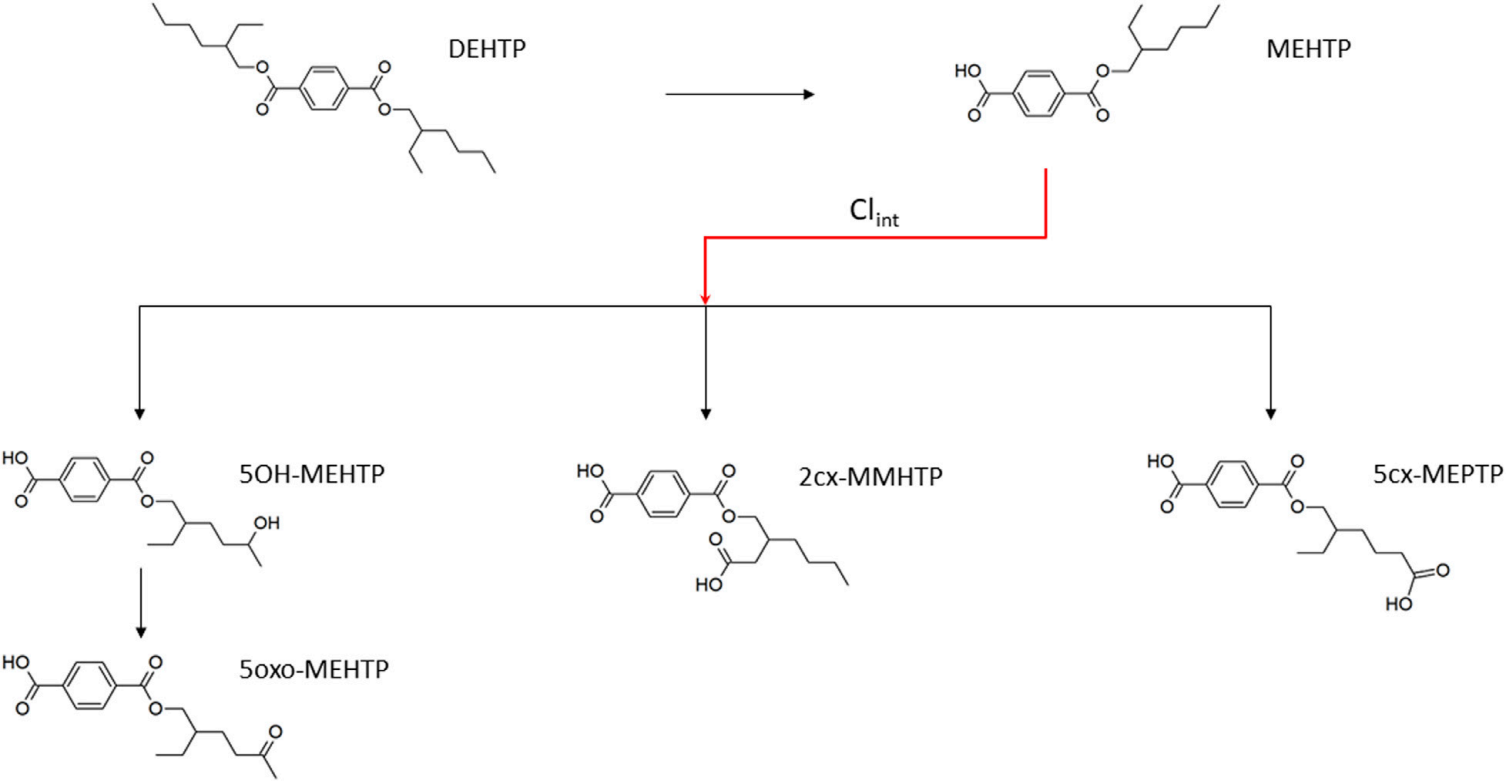
Metabolic pathway of DEHTP to the specific, side-chain-oxidized monoesters measured in the controlled human exposure study of Lessmann et al. (2016) and simulated using the PBPK model. The intrinsic clearance, Cl_int_ for the biotransformation of MEHTP to the three urinary metabolites is shown by the red arrow. Cleavage to the unspecific metabolite terephthalic acid (TPA), and phase II metabolism (conjugation with, e.g., glucuronic acid) not shown for simplification.

The very high lipophilicity of DEHTP resulted in the formation of an insoluble film on the surface of the reaction medium which precluded the measurement of *in vitro* clearance which is consistent with previous studies (McNally et al., 2019; McNally et al., 2021). Therefore, only the measurement of *in vitro* clearance of MEHTP was possible (Figure 1). *In vitro* incubations, the determination of *in vitro* half-life, *in vitro* intrinsic clearance and the calculation of *in vivo* clearance were identical to previous studies and are described therein (McNally et al., 2019; McNally et al., 2021).

The NADPH regenerating system consisted of the following final concentrations: 1.3 mM NADP^+^; 3.3 mM glucose-6-phosphate; 5 mM magnesium chloride; 0.4 U/ml glucose-6-phosphate dehydrogenase; 50 mM phosphate buffer (pH 7.4). Final microsomal protein concentration was 0.5 mg/ml. Incubations were performed in polypropylene tubes and pre-warmed reaction mixtures were started by addition of substrate dissolved in acetonitrile. The final acetonitrile concentration was less than 1% and, typically, a substrate concentration of 10 µM was used (initial investigations were performed to check solubility in the reaction mixture). Incubations were conducted in a water bath at 37°C. At the time points chosen for measurement, tubes were mixed by inversion and an aliquot removed and quenched by adding to an equal volume of ice-cold methanol followed by centrifugation to precipitate the protein as a pellet. The supernatant was removed for analysis. Three replicates were sampled at each time point. Control incubations consisted of a reaction mix excluding glucose-6-phosphate dehydrogenase (for evaluation of non-specific binding) and reaction mix excluding microsomes (for evaluation of substrate stability).

The method of Jones and Houston (2004) was used to determine the *in vitro* half-life of substrate depletion. At least three independent incubations were performed, and results were assessed visually for reproducibility. However, due to differences in sampling time points between experiments, results from individual incubations were not combined.

#### Calculation of in vitro intrinsic clearance

The *in vitro* intrinsic clearance for MEHTP, *CL*_*in vitro*_ (ml min^−1^ mg^−1^ microsomal protein) in human hepatic microsomes was calculated using the half-life (*T*_*½*_) derived from the decay constant (*k*) using the following equations (Obach 1997):

$$in\;vitroT_{1/2}=\frac{\ln\left(2\right)}{k}$$ (1)

$${CL}_{in\;vitro=\frac{\ln\left(2\right)}{in\;vitroT_{1/2}}\times \frac{mlincubation}{mgmicrosomes}}$$ (2)

Where, *ml incubation* is the volume (ml) of the incubation medium and *mg microsomes* is the mass (mg) of microsomes in the incubation medium.

#### Calculation of in vivo clearance

The intrinsic hepatic clearance CL_int_H_ (L h^−1^) was calculated using the following formula adapted from Obach (1999):

$${CL}_{\mathrm{int}\_H}={CL}_{in\;vitro}\times MPY\times Vli\times 60$$ (3)

Where, *MPY* is the microsomal protein yield per g liver (mg g^−1^), *Vli* is mass of the liver (g) and the 60 converts from minutes to hours.

Whole liver plasma clearance *CL*_*H*_ (L h^−1^) was calculated assuming the well-stirred model of hepatic clearance taking into account the unbound fraction in plasma, *fu* and the red blood cells to plasma ratio, C_RBC_/C_P_, using the following equation (Yang et al., 2007):

$${CL}_{H}=Q_{H}∙fu∙\frac{{CL}_{\mathrm{int}\_H}}{Q_{H}+fu∙{CL}_{\mathrm{int}\_H}/\left(C_{RBC}/C_{p}\right)}$$ (4)

Where, *Q*_*H*_ (L h^−1^) is the blood flow to the liver as a proportion of cardiac output.

The intrinsic gut clearance CL_int_gut_ was calculated similarly as described for hepatic clearance but substituting *MPY*_*gut*_ and *Vgu* for *MPY* and *Vli*, respectively, in Eq.4. The resulting calculated CL_int_gut_ was used in place of CL_int_H_ for calculation of CL_gut_.

#### Prediction of log P_ow_ and tissue: Blood partition coefficients and plasma fraction unbound

The tissue:blood PCs and unbound fractions in plasma were calculated from the logarithm of the octanol–water PC, Log P_ow_ as described in McNally, et al. (2019) and McNally et al. (2021). The Log P_ow_ for DEHTP and MEHTP were calculated using the ACDLogP algorithm (Mannhold et al., 2009) implemented in the ACD/ChemSketch 2019.1.0^3^ software (Table 1). Two tissue-composition-based algorithms for the calculation of tissue:blood PCs were used. The method of Poulin and Haddad (2012), developed for the prediction of the tissue distribution of highly lipophilic compounds, defined as chemicals with a Log P_ow_ > 5.8, was used for DEHTP (Table 1). The method of Schmitt (2008), developed to predict the tissue distribution of chemicals with Log P_ow_ < 5.17, was used to predict the PCs of the monoester, MEHTP (Table 1). The algorithm of Poulin and Haddad (2012) was implemented as a Microsoft^®^ Excel Add-in whereas a modified version of the algorithm of Schmitt (2008) was available within the httk: R Package for High-Throughput Toxicokinetics (Pearce et al., 2017). Where the tissue-composition-based algorithms did not provide a tissue:blood PC for a particular compartment, the value from a surrogate organ or tissue with similar blood perfusion rate (i.e., could be lumped within the rapidly or slowly perfused compartments) was assumed. These are presented in italicised text with the surrogate organ or tissue in brackets Table 1.

**TABLE 1:** Tissue:blood partition coefficients and plasma fraction unbound predicted using Log P_ow_.

	DEHTP	MEHTP
Log Po:w	9.54	5.84
Tissue:blood partition coefficient		
Adipose	47.2	20.3
Kidney	3.7	12.2
Liver	5.9	5.9
Muscle	3.3	3.3
Blood cells	3.0	3.0
Gut	7.4	7.4
Spleen	3.7	3.7
*Stomach* *^1^* *(gut)*	7.4	7.4
*Rapidly Perfused (spleen)*	3.7	3.7
*Slowly Perfused (muscle)*	3.3	3.3
		
Plasma Fraction Unbound	0.000158	0.007175

^1^Compartments in italics have surrogate values from another organ compartment. The corresponding surrogate organ compartment is in parentheses.

The fraction unbound (*fu*) was calculated from *log((1-fu)/fu)* with the following equation:

$$fu=\frac{1}{10^{x}+1}$$ (5)

Where, x=0.4485logP−0.4782

When *x* is the equation for the prediction of *fu* for a chemical with a predominantly uncharged state at pH 7.4 (Lobell and Sivarajah, 2003) (Table 1).

#### Calculation of fraction metabolised

The three metabolites, 1-Mono-(2-carboxyl-methyl-hexyl) benzene1,4-dicarboxylate (2cx-MMHTP), 1-Mono-(2-ethyl -5-carboxyl-pentyl) benzene-1,4-dicarboxylate (5cx-MEPTP) and 1-Mono-(2-ethyl-5-hydroxy-hexyl) benzene-1,4-dicarboxylate (5OH-MEHTP) that are directly bio-transformed from MEHTP only were simulated (Figure 1). However, the amount of 1-Mono-(2-ethyl-5-oxo-hexyl) benzene-1,4-dicarboxylate (5oxo-MEHTP) produced was included in the total amount of metabolites to calculate the proportions of the measured metabolites. The proportion of DEHTP metabolised to MEHTP and from MEHTP to 2cx-MMHTP, 5cx-MEPTP and 5OH-MEHTP for each volunteer was estimated by expressing all the biological monitoring (BM) data (MEHTP, 2cx-MMHTP, 5cx-MEPTP, 5OH-MEHTP, 5oxo-MEHTP) in moles and dividing the individual amounts of 2cx-MMHTP, 5cx-MEPTP, 5OH-MEHTP by the total amount of all metabolites (5oxo-MEHTP was not simulated because this compound is a metabolite of 5OH-MEHTP) (Table 2).

**TABLE 2:** Volunteer specific parameters.

	A	B	C
Body weight (kg)	94	85	95
Dose (mg kg^−1^)	0.555	0.614	0.549
Fraction Metabolised from DEHTP			
to MEHTP	0.763	0.860	0.860
Fraction Metabolised from MEHTP			
to 5OH-MEHTP	0.024	0.013	0.013
to 2cx-MMHTP	0.004	0.003	0.003
to 5cx-MEPTP	0.204	0.116	0.116

#### Biological monitoring data

The biological monitoring (BM) data from the volunteer study of Lessmann et al. (2016) were simulated in this investigation. Briefly, three healthy male volunteers (aged 32–45; body weight 85–95 kg) received an oral dose of approximately 50 mg (weighted exactly) DEHTP dissolved in 1 ml ethanol in a chocolate coated waffle cup containing water. The resulting dosages amounted to 0.549–0.614 mg/kg body weight (Table 2). The volunteers did not have any occupational exposure to DEHTP. The volunteers donated 20, 22, and 23 individual urine samples over a 48-h period with total urine volumes of 3620, 4050, and 5590 ml, respectively.

The concentrations of 2cx-MMHTP, 5cx-MEPTP, 5OH-MEHTP (mg/l) were extracted from the dataset. The rates of deposition of metabolite into the bladder (mg/h) were calculated based on the concentrations (mg/l), the volume of the urine void (l) and the time between successive voiding events. This rate represents an average rate of deposition since the previous urination event and renders the trends in urine data more clearly (Nehring et al., 2020). The derived rate was associated with the mid-point between the two voiding events.

#### The PBPK model

An existing human PBPK model for DPHP (McNally and Loizou, 2023) was adapted for studying the absorption, distribution, metabolism, and elimination of DEHTP following single oral doses on the basis that: a) the key mechanisms captured in the PBPK model for DPHP appeared to also be relevant for DEHTP; b) a comparison of concentration-time profile data from urine voids on second order metabolites of DPHP and DEHTP showed similar shape profiles to the data.

**FIGURE 2:**
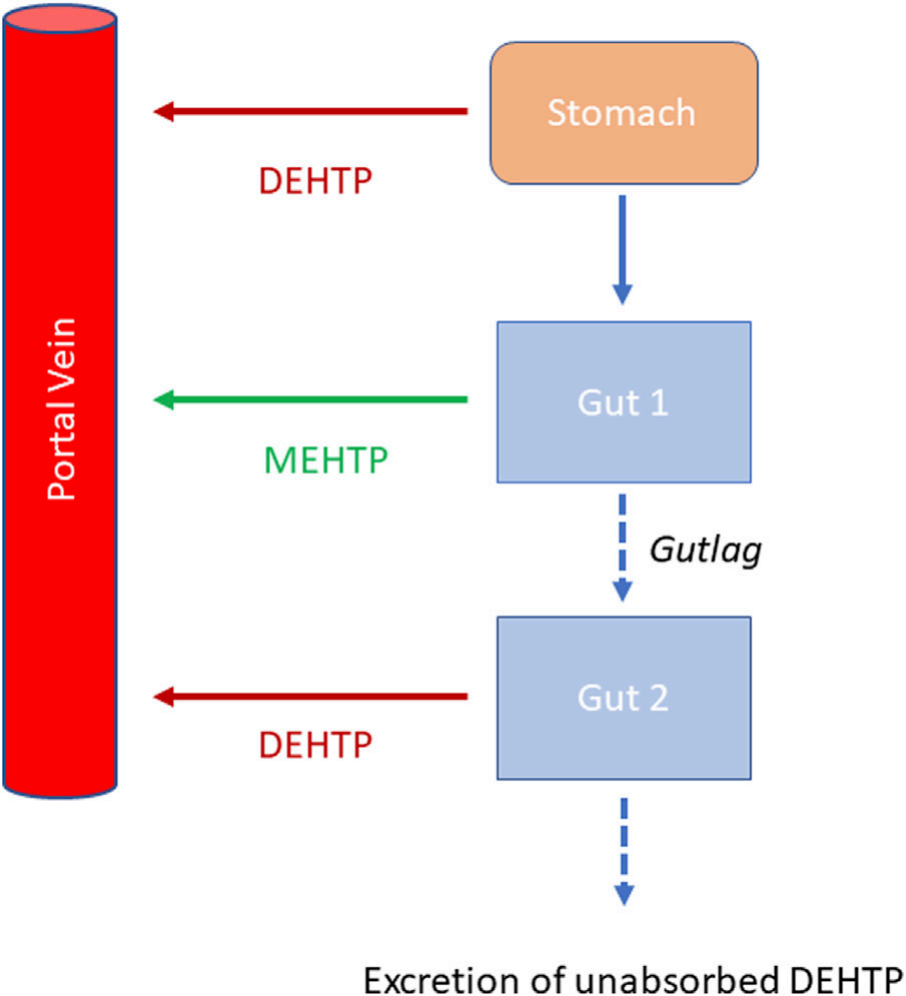
Schematic of the simplified stomach and two-stage gut compartments.

Briefly, the model for DEHTP described two distinct uptake processes and allowed for a fraction to pass directly through the gut and be ultimately eliminated in faces. The first uptake process was into blood. The model included a simplified two-stage intestine compartment to describe absorption from the stomach and gastro-intestinal (GI) tract. Uptake of DEHTP into venous blood from the stomach, metabolism of DEHTP to MEHTP and uptake of MEHTP into venous blood was ascribed to the first intestine compartment, and finally uptake of DEHTP into venous blood was ascribed to the second-phase intestine compartment. A parameter, *Gutlag* was included to represent a delay in transport of DEHTP through the intestine transiting from the first to second intestine compartment (Figure 2). A fraction of MEHTP absorbed through the first intestine compartment was coded as being unavailable for first pass metabolism in the liver following uptake into venous blood—this represents incomplete binding. The second important uptake mechanism of DEHTP was into the lymphatic system. Uptake of DEHTP *via* the lacteals in the intestine and subsequent entry into venous blood after bypassing the liver was described. The assumption that DEHTP, like DEHP, binds like lipid to lipoproteins (Griffiths et al., 1988) which are formed in enterocytes and transported in the lymph to enter the venous blood *via* the thoracic duct, justified the inclusion of a lymph compartment (Kessler et al., 2012). The fractions of dose entering venous blood, the lymphatic system and passing straight through the gut summed to unity.

Metabolism of DEHTP to MEHTP was ascribed to the liver and a section of the intestine (Gut 1, Figure 2). The model for DEHTP additionally encoded the transport process of enterohepatic recirculation. Uptake of DEHTP from the liver into bile was modelled as a first order uptake process with a delay (to represent transport from liver to gut) before DEHTP appeared in the small intestine where it was available for reabsorption.

**FIGURE 3:**
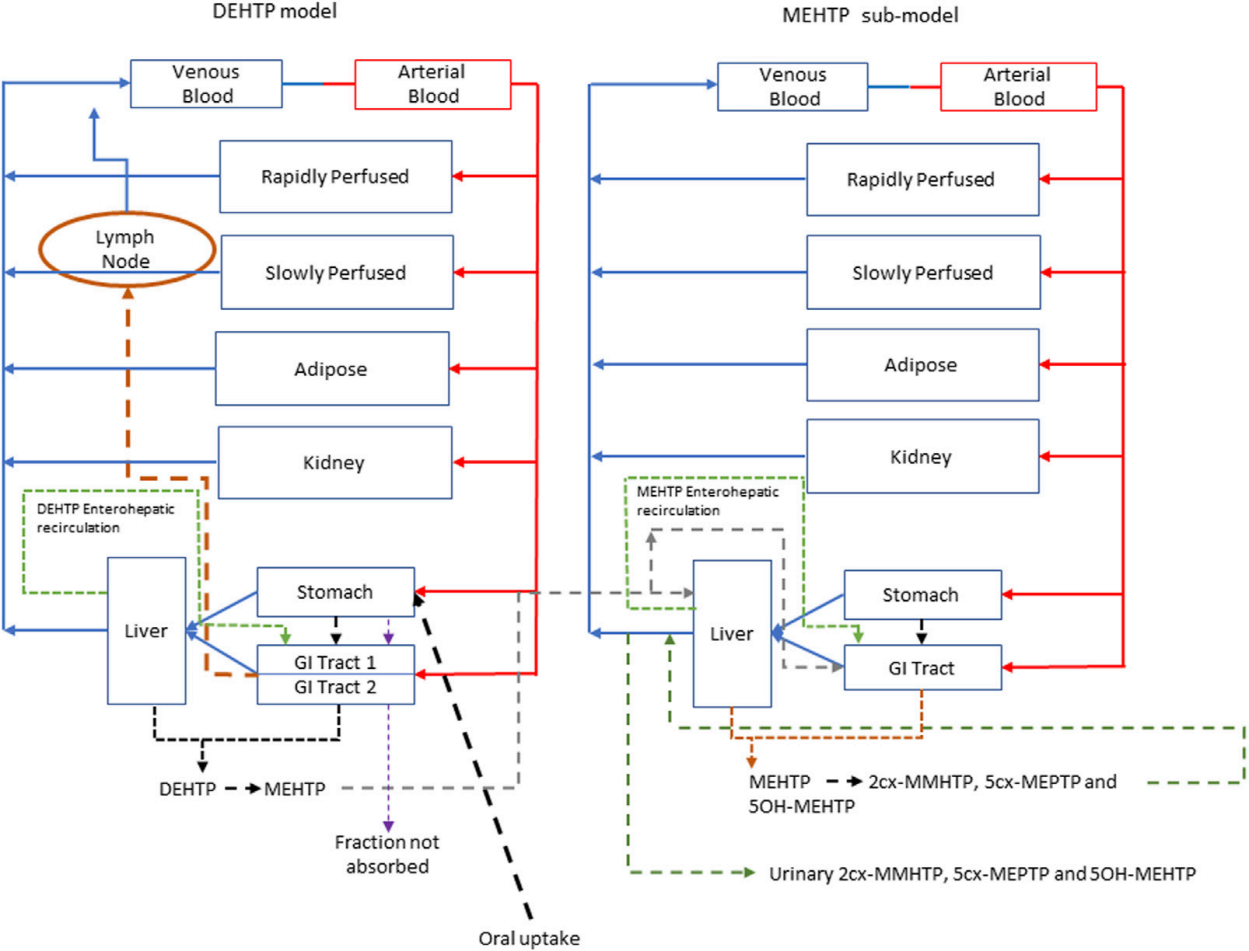
A schema of the model for DEHTP and sub-model for MEHTP. The main model contained a lymphatic compartment (- - - -) which received a portion of oral dose from the stomach and GI tract. Urinary excretion of metabolites was described with a first-order elimination rate constant ascribed to the sub-model. See Figure 2 for an expanded schematic of the modified stomach and GI tract compartments.

The model had a stomach and (the two-phase) intestine draining into the liver and systemically circulated to adipose, kidney, blood (plasma and red blood cell) and slowly and rapidly perfused compartments (Figure 3). Protein binding was described in arterial blood, with only the unbound fraction of DEHTP available for distribution to organs and tissues and metabolism.

A sub-model was coded to describe the kinetics of MEHTP. As described above, metabolism of DEHTP to MEHTP was coded in the first intestinal compartment and the liver, therefore models for DEHTP and MEHTP were connected at these nodes in the model. Metabolism of MEHTP was coded in the liver alone. The MEHTP sub-model had a stomach and (single-phase) intestine draining into the liver and systemically circulated to adipose, kidney, blood (plasma and red blood cell) and slowly and rapidly perfused compartments (Figure 3). Elimination of MEHTP from the kidney was described with a first-order elimination rate. As with the DEHTP model, binding was described in arterial blood.

To make use of biological monitoring data on three metabolites of MEHTP (5OH-MEHTP, 2cx-MMHTP and 5cx-MEPTP) it was necessary to include the elimination of these substances into urine. A particular difficulty in the specification of a mathematical model was that terephthalic acid (TPA) is a major, although non-specific, metabolic product of DEHTP; (i.e., there are other dietary sources) (Silva et al., 2015). There is a non-specific metabolic pathway resulting in the production of TPA directly from DEHTP, from metabolism of MEHTP or from further metabolism of second order metabolites. Whilst TPA was not measured in the biomonitoring data of (Lessmann et al., 2016) it was necessary to account for a large fraction of administered DEHTP being eliminated in urine as TPA. A simplified representation of these downstream metabolites in the model was therefore assessed as being suitable for the aims of modelling. The fraction of MEHTP eliminated in urine as TPA (either as a direct metabolite or through further metabolism of second order metabolites) was estimated to be 30%–60% (Dr. Rainer Otter, (Members of European Plasticisers), personal communication). Fractions of MEHTP that were *metabolised to, and eliminated from the blood as* 5OH-MEHTP, 2cx-MMHTP and 5cx-MEPTP were coded with appropriate limits for these fractions based on the BM data from Lessmann et al. (2016) (Table 2) adjusted for TPA (which accounted for between 30% and 60% of all MEHTP). Consequently, the uncertainty in these respective fractions characterised by prior distributions in calibration was substantially wider than the BM data (Table 2) would suggest. First order elimination constants described the removal of these respective fractions from blood and into urine. The kinetics of these second order metabolites were thus described using six parameters in all. The model did not describe the distribution of these metabolites to organs and tissues.

The model code is available in Supplementary Materials.

### Statistical analysis

#### Parameter distributions

Probability distributions for uncertainty and sensitivity analysis of the final PBPK model are listed in Table 3. Distributions for anatomical and physiological parameters were obtained from PopGen the freely available web-based application (McNally et al., 2014). A population of 10,000 Caucasian males was generated. The range of ages, heights and body weights supplied as input to PopGen were chosen to encompass the characteristics of the volunteers who participated in the human volunteer study of Lessmann et al. (2016). Organ masses and blood flow ranges were modelled by normal or log-normal distributions as appropriate with parameters estimated from the sample and truncated at the 5th and 95th percentiles.

**TABLE 3:** Physiological and kinetic default values used in PBPK model and probability distributions applied for uncertainty and sensitivity analyses.

Physiological Parameters	Abbreviation	Default Value	Distribution
			
Body weight (kg)	BW	89	N^1^(49, 130)
% BW			
Total vascularised tissues	VT	0.95	-
Liver	VLiC	3.09	N (3.09, 0.8)
Kidney	VKic	0.58	N (0.58, 0.15)
Fat	VFaC	19.5	LN (3.42, 0.43)
Gut	VGuC	1.50	N (1.50, 0.17)
Stomach	VStC	0.22	N (0.22, 0.07)
Slowly perfused tissue	VSpdC	60.7	N (60.7, 9.4)
Rapidly perfused tissue	VRpdC	3.71	N (3.7, 0.26)
Blood	VBldC	5.0	N ((5.0,1.0)
			
Cardiac output (L h^−1^ kg^−1^ BW)	QCC	14	N (13.8, 2.5)
			
% Cardiac output			
Liver	QHepartC	6.0	N (6.89, 0.52)
Kidney	QKiC	20.0	N (20, 3)
Fat	QFaC	5.0	N (5.3, 0.3)
Gut	QGuC	14.9	N (14.9, 0.9)
Stomach	QStC	1.1	N (1.1, 0.08)
Slowly perfused tissue	QSpdC	27.0	N (28.7, 1.91)
Rapidly perfused tissue	QRpdC	22.0	N (23.1, 2.78)
Blood:tissue partition coefficients			
*DEHTP*			
Plasma	Pbab	15.5	U (1,30)
Adipose	Pfab	47.2	U (32, 125)
Liver	Plib	5.89	U (1,50)
Kidney	Pkib	3.7	U (3, 12)
Red blood cells	Prbcb	3.0	U (1, 10)
Gut	Pgub	7.4	U (1,50)
Stomach	Pstb	3.7	U (2, 8)
Rapidly Perfused	Prpdb	3.7	U (2, 8)
Slowly Perfused	Pspdb	3.3	U (2,8)
*MEHTP*			
Plasma	PbaM	25.23	U (1, 50)
Adipose	PfaM	20.3	U (15, 60)
Liver	PliM	5.9	U (1, 30)
Kidney	PkiM	12.2	U (1, 30)
Red blood cells	PrbcM	6.67	U (3, 12)
Gut	PguM	7.4	U (1, 30)
Stomach	PstM	7.4	U (12, 50)
Rapidly Perfused	PrpdM	3.7	U (6, 24)
Slowly Perfused	PspdM	3.3	U (4, 15)
			
Metabolic Clearance (minutes)			
*In vivo* half-life DEHTP	T_½DEHTP_	3^2^	HN(10)
*In vitro* half-life MEHTP	T_½MEHTP_	30.54	N (30.54, 2.39)
*In vivo* DEHTP gut half-life	T_½DEHTP_gut_	60^3^	N (30, 10)
Elimination (liver to bile) (h^−1^)			
DEHTP	k1_DEHTP_liver	1	HN(2)
Microsomal protein yield (mg g^−1^)			
Hepatic	MPY	34^4^	N (34, 10)
Gut	MPY_gut_	3.34^5^	N (3.34, 1^e^67)

Fraction Bound in plasma (proportion)	Abbreviation	Default Value	Distribution
DEHTP	FBDEHTP	0.9998	U (0.8, 1.0)
MEHTP	FBMEHTP	0.992	U (0.8, 1.0)
			
Gastric emptying (h^−1^)^6^			
Maximum	k_(max)_	10.2	U (5.1, 20.4)
Minimum	k_(min)_	0.005	U (0.0025, 0.01)
			
Absorption (h^−1^)			
Gut	k_Ga_	25.1	U (12.55, 50.2)
Time taken to consume dose (h)	DRINKTIME	0.25	U (0.125, 0.5)
Absorption in Stomach	BELLYPERM	0.685	U (0.0, 7.5)
Absorption in GI Tract 1	GIPERM1	0.2	U (0.0, 10.0)
Absorption in GI Tract 2	GIPERM2	0.2	U (0.0, 10.0)
Absorption in Lymph *via* stomach	BELLYPERMLymph	0.685	U (0.34, 0.99)
Absorption in Lymph *via* GI Tract	GIPERMLymph	5.1	U (2.6, 7.6)
Absorption into blood from lymph	K1Lymph	0.2	U (0.0, 3.0)
Fraction of dose taken up into liver	FRACDOSEHep	0.1	U (0, 0.7)
Fraction of dose taken up into lymphatic system	FracDOSELymph	0.05	U (0, 0.3)
			
Delays (h^−1^)			
Transportation delay in gut	Gutlag	1	U (0.01, 5)
Delay associated with lymphatic uptake	LymphLag	1	(0.01, 7)
			
Uptake (% of ingested dose)			
Fraction of MEHTP metabolised to 2cx	FracMetab2cx	1.0	U (0.5, 1.5)
Fraction of MEHTP metabolised to 5cx	FracMetab5cx	42.5	U (30.0, 55.0)
Fraction of MEHTP metabolised to OH	FracMetabOH	6.5	U (4.5, 8.5)
			
Fraction unavailable for first pass metabolism (fraction of dose)			
MPHP in gut	EscapeFracgut	0.5	U (0,1)
MPHP in liver	EscapeFracliver	0.5	U (0,1)
			
Urinary elimination rate (h^−1^)			
5OH-MEHTP	K1_MOH	0.1	U (0.0, 5.0)
2cx-MMHTP	K1_2cx	0.1	U (0.0, 5.0)
5cx-MEPTP	K1_5cx	0.1	U (0.0, 5.0)

^1^Distributons, N = normal, LN, lognormal; HN, Half-nomal, U = uniform.

^2^ Estimated.

^3^ Estimated.

^4^(Howgate et al., 2006; Barter e al., 2007).

^5^(Pacifici et al., 1988; Soars, et al., 2002).

^6^(Loizou ad Spendiff, 2004).

The various delay terms and uptake and elimination rates were assigned uniform distributions. The upper and lower bounds in Table 3 were refined during the model development process. The tabulated values are based upon expert judgement and represent conservative yet credible bounding estimates.

#### Uncertainty and sensitivity analysis

McNally et al. (2021) describe an interactive approach for development and testing of the human PBPK model for DPHP using techniques for uncertainty and sensitivity analysis to study the behaviour of the model and the key parameters that drove variability in the model outputs. The principal techniques used for model evaluation were Latin Hypercube Sampling (LHS) to evaluate the qualitative behaviour of the model, and a two-phased sensitivity analysis consisting of elementary effects screening and a variance-based sensitivity analysis to identify the important uncertain parameters in the model to be refined in calibration. McNally and Loizou (2023) describe additional analysis for a refined PBPK model of DPHP. Given that the structure of PBPK models for DPHP and DEHTP for parent chemical and first metabolite are almost identical (the DPHP and DEHTP models only differing in the molecular weights and chemical specific parameters), a bespoke analysis to study the behaviour of the PBPK model for DEHTP, and the subset of sensitive parameters to be tuned in calibration, was not considered to be necessary; results from such work are not presented. However, testing of the PBPK model for DEHTP, sufficient to verify the coding and the ability to capture the trends seen in the BM data of (Lessmann et al., 2016), has been undertaken. Thirty-four parameters (Tables 4, 5) were taken forward into calibration.

**TABLE 4:** Global prior and posterior distributions.

Parameter	Median (95% interval)	
	Prior	Posterior
FB_DEHTP	0.901 (0.805, 0.995)	0.858 (0.802, 0.952)
FB_MEHTP	0.901 (0.805, 0.995)	0.858 (0.802, 0.952)
DEHTP_GUT_half_life	30.13 (10.67, 49.37)	33.60 (17.80, 50.51)
DEHTP_half_life	6.62 (0.322, 22.34)	2.71 (0.10, 10.85)
Pbab	15.5 (1.785, 29.27)	24.26 (10.71, 29.72)
Pgub	25.23 (2.12, 48.70)	41.50 (17.65, 49.55)
Plib	25.23 (2.12, 48.70)	14.81 (1.55, 45.92)
PbaM	25.23 (2.12, 48.70)	37.53 (17.30, 49.58)
PliM	15.5 (1.785, 29.27)	18.64 (4.91, 28.99)
PguM	15.5 (1.785, 29.27)	12.18 (1.41, 29.29)
K1_2cx	2.49 (0.12, 4.89)	3.58 (1.62, 4.87)
K1_MOH	2.49 (0.12, 4.89)	2.28 (1.18, 3.62)
K1_5cx	2.49 (0.12, 4.89)	3.91 (1.68, 4.91)
escapeFracgu	0.49 (0.026, 0.976)	0.286 (0.014, 0.921)
escapeFracli	0.49 (0.026, 0.976)	0.022 (0.0005, 0.11)
$\sigma_{2cx\_U}$	0.67 (0.032, 2.20)	0.00057 (0.00044, 0.00074)
$\sigma_{OH\_U}$	0.67 (0.032, 2.20)	0.0059 (0.0047, 0.0075)
$\sigma_{5cx\_U}$	0.67 (0.032, 2.20)	0.023 (0.018, 0.030)

#### Calibration

Calibration is the process of tuning a subset of model parameters such that the discrepancy between model predictions and comparable measured data is minimised. This is achieved through the specification of an error model that links predictions to measurements. A Bayesian approach was followed (McNally et al., 2012) for calibration in this work since this allows the uncertainty in parameters, and thus uncertainty in the concentration response predictions from the PBPK model, to be explicitly quantified.

A Bayesian approach requires the specification of a joint prior distribution for the parameters under study. It is necessary to distinguish between two classes of parameters: global parameters which are common to all individuals (appropriate for various constants and physicochemical properties such as partition coefficients etc.); and local parameters, which vary between individuals (suitable for accounting for variability in physiology and modelling the participant specific uptake of DEHTP etc.). These two classes are denoted by the vectors θ and $\omega_{j}$ respectively, where the subscript *j = 1 … 3,* denotes the participant. A prior distribution for each sensitive global parameter was specified through the distributions provided in Table 3. A prior distribution for each individual (three copies in all) was specified for each of the local parameters. These distributions are also provided in Table 3. A median and 95% interval for global and local parameters is provided in Tables 4 (global) and Table 5 (locals) respectively. Insensitive parameters were fixed at baseline values (Table 3) during calibration.

**TABLE 5:** Local posterior distributions.

Parameter	Prior	Ind 1	Ind 2	Ind 3
FracMetabMOH	0.065 (0.046, 0.084)	0.052 (0.045, 0.066)	0.055 (0.046, 0.066)	0.081 (0.072, 0.084)
FracMetab2cx	0.010 (0.005, 0.015)	0.009 (0.007, 0.011)	0.011, (0.010, 0.013)	0.008 (0.007, 0.009)
FracMetab5cx	0.425 (0.306, 0.543)	0.437 (0.366, 0.544)	0.507 (0.435, 0.548)	0.314 (0.301, 0.354)
K1_DEHTP_Liver	2.060 (0.089, 6.83)	0.554 (0.028, 2.91)	0.196 (0.006, 1.58)	1.80 (0.26, 4.74)
FracDOSEHep	0.354 (0.018, 0.681)	0.290 (0.187, 0.401)	0.031 (0.021, 0.07)	0.142 (0.081, 0.216)
BELLYPERM	3.72 (0.24, 7.30)	6.37 (2.54, 7.44)	3.01 (0.22, 9.55)	3.57 (0.49, 7.29)
GIPERM1	4.98 (0.28, 9.74)	5.61 (0.39, 9.84)	2.666 (0.162, 9.61)	3.63 (0.51, 9.68)
GIPERM2	15.17 (0.72, 29.20)	8.69 (0.31, 29.10)	14.84 (1.41, 29.10)	9.01 (0.488, 28.68)
Gutlag	1.98 (0.11, 3.90)	3.52 (1.97, 3.86)	2.05 (1.32, 2.79)	1.94 (0.36, 3.98)
FracDoseLymph	0.152 (0.008, 0.293)	0.058 (0.004, 0.253)	0.26 (0.224, 0.296)	0.09 (0.015, 0.159)
Lymphlag	3.00 (1.09, 4.90)	2.55 (1.19, 4.73)	4.72 (4.52, 4.93)	3.65 (1.04, 4.66)
K1_Lymph	1.53 (0.11, 2.92)	1.25 (0.285, 2.92)	1.46 (0.734, 2.85)	2.33 (0.81, 2.95)
MPY	34.0 (14.54, 53.77)	45.44 (29.42, 61.60)	46.28 (32.26, 64.35)	41.18 (25.80, 58.11)
MPYgu	3.92 (0.58, 7.79)	3.35 (0.97, 6.39)	2.66 (0.27, 6.12)	3.44 (0.40, 7.02)
VBldC	0.05 (0.031, 0.070)	0.049 (0.031, 0.070)	0.05 (0.029, 0.069)	0.049 (0.029, 0.067)
VliC	0.03 (0.011, 0.05)	0.036 (0.023, 0.048)	0.041 (0.029, 0.049)	0.039 (0.024, 0.049)
VguC	0.015 (0.010, 0.020)	0.016 (0.011, 0.019)	0.015 (0.011, 0.019)	0.014 (0.010, 0.018)
VkiC	0.0058 (0.0028, 0.007)	0.006 (0.004, 0.008)	0.006 (0.004, 0.008)	0.006 (0.003, 0.008)
QguC	0.150 (0.089, 0.21)	0.14 (0.091, 0.207)	0.185 (0.141, 0.231)	0.168 (0.112, 0.227)

The second facet of model specification is the statistical error model. The final calibration model utilised BM data from the three volunteers and three specific outputs were formally compared within the error model. The rates of deposition of 5OH-MEHTP, 2cx-MMHTP and 5cx-MEPTP (mg/h) into the bladder (*RUrine MEHTP*, *RUrine OH, RUrine 2cx* and *RUrine 5cx*) were computed from the raw data of Lessmann et al. (2016) as described earlier, and compared with equivalent predictions extracted from the PBPK model through Equations 6–8.

The terms ${RUrineOH}_{ij}$ ${RUrine2cx}_{ij}$ and ${RUrine5cx}_{ij}$ denote measurement i (at time $t_{i}$ ) for individual j (for j *in 1:3)* for the three respective model outputs, whereas $\mu_{OH\_U}{\left(\theta ,\omega_{j}\right)}_{ij}$ , $\mu_{2cx\_U}{\left(\theta ,\omega_{j}\right)}_{ij}$ and $\mu_{5cx\_U}{\left(\theta ,\omega_{j}\right)}_{ij}$ , denote the predictions from the PBPK model corresponding to parameters ( $\theta ,\omega_{j}$ ). Normal distributions, truncated at zero were assumed for all three relationships, where $\sigma_{OH\_U}$ $\sigma_{2cx\_U}$ and $\sigma_{5cx\_U}$ denote the respective error standard deviations,

$${RUrineOH}_{ij}∼\;N\left(\mu_{OH\_U}{\left(\theta ,\omega_{j}\right)}_{ij},\sigma_{OH\_U}\right)\left[0,\infty \right]$$ (6)

$${RUrine2cx}_{ij}∼\;N\left(\mu_{cx\_U}{\left(\theta ,\omega_{j}\right)}_{ij},\sigma_{cx\_U}\right)\left[0,\infty \right]$$ (7)

$${RUrine5cx}_{ij}∼\;N\left(\mu_{cx\_U}{\left(\theta ,\omega_{j}\right)}_{ij},\sigma_{cx\_U}\right)\left[0,\infty \right]$$ (8)

Weakly informative, half-normal prior distributions with standard deviations of 1 were assumed for the three standard deviation parameters in Equations 2–4.

Inference for the model parameters was made using Markov chain Monte Carlo (MCMC) implemented in MCSim (see Software). Inference for model parameters in the final calibration model was made using thermodynamic integration (TI) as described in (Bois et al., 2020). A single chain of 150,000 iterations was run with every 10th retained.

#### Software

The PBPK model was written in the GNU MCSim^4^ language and run using the RStudio Version 1.3.1093^5^. PBPK models were solved using the deSolve package of R^6^. The DiceDesign package of R was used for generating Latin Hypercube designs. GSA of model outputs (through elementary effects screening and eFAST) were conducted using the Sensitivity package of R. The reshape2 package of R was used for reshaping of data for plotting and other processing of results. MCMC was undertaken using the thermodynamic integration (TI) option within GNU MCSim. All plots were created using R and the ggplot2^7^ package.

## Results

### Evaluation of model fit

**FIGURE 4:**
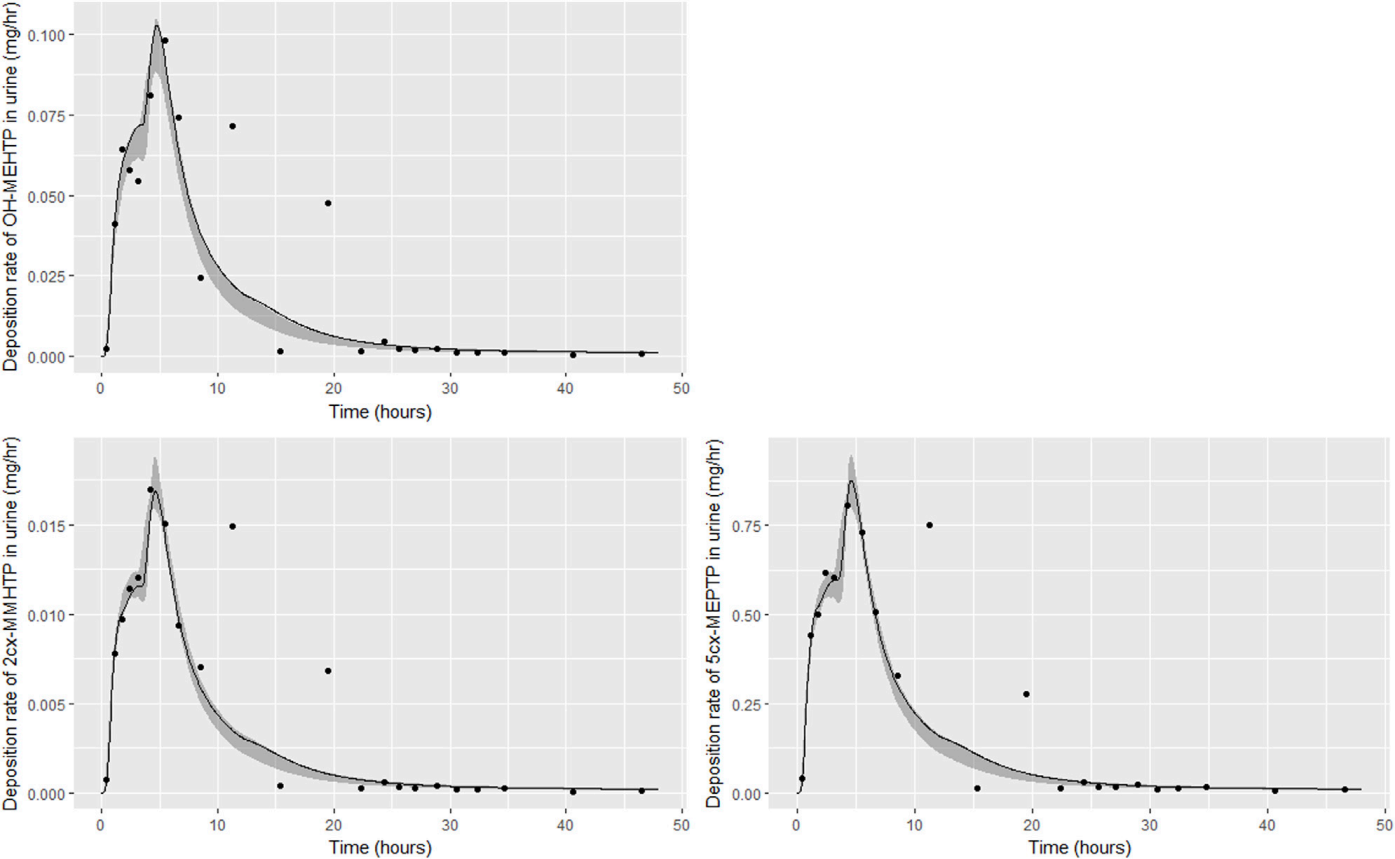
Fit of the calibrated model to urinary excretion data from individual **(A)**. Panel **(A)** OH-MEHTP (mg/h); **(B)** 2cx-MMHTP (mg/h); **(C)** 5cx-MEPTP (mg/h). The central estimates indicated in plots correspond to the posterior mode whereas the shaded regions represent 95% intervals for the respective curves.

**FIGURE 5:**
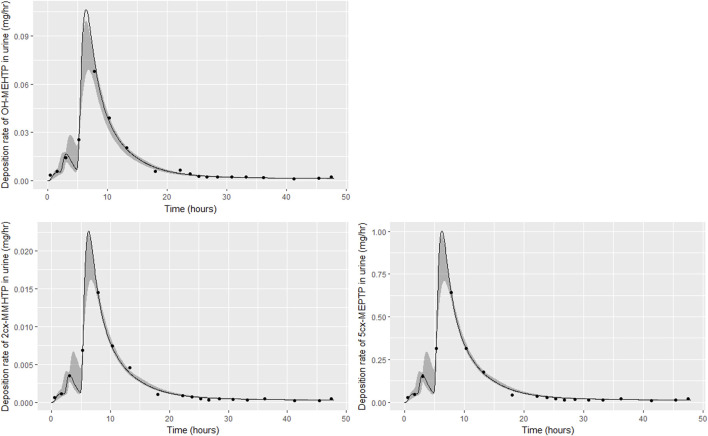
Fit of the calibrated model to urinary excretion data from individual **(B)**. Panel **(A)** OH-MEHTP (mg/h); **(B)** 2cx-MMHTP (mg/h); **(C)** 5cx-MEPTP (mg/h). The central estimates indicated in plots correspond to the posterior mode whereas the shaded regions represent 95% intervals for the respective curves.

**FIGURE 6:**
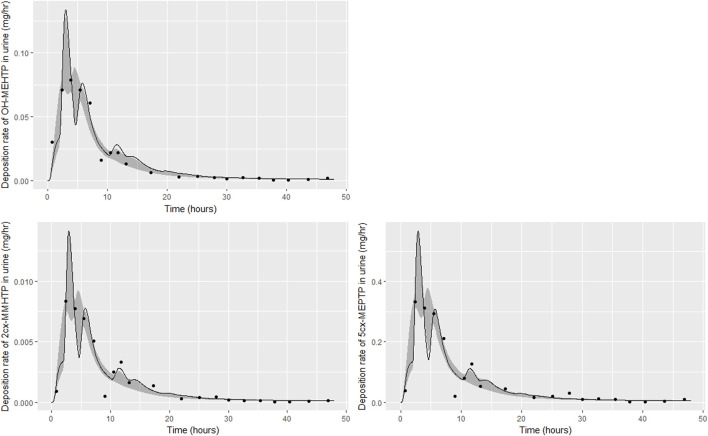
Fit of the calibrated model to urinary excretion data from individual **(C)**. Panel **(A)** OH-MEHTP (mg/h); **(B)** 2cx-MMHTP (mg/h); **(C)** 5cx-MEPTP (mg/h). The central estimates indicated in plots correspond to the posterior mode whereas the shaded regions represent 95% intervals for the respective curves.

Summary statistics based upon the retained sample (posterior median and a 95% credible interval) for the 15 global and 19 local (volunteer specific) parameters are provided in Tables 4, 5 respectively. Table 6 shows a comparison of the measured and model predicted 48-h excretions of the three modelled metabolites. The fit of the calibrated model is shown in Figures 4–6 for individuals A, B and C respectively. The three panels in each figure correspond to A) deposition of OH-MEHTP in urine (mg/h); B) deposition of 2cx-MMHTP in urine (mg/h); C) deposition of 5cx-MEPTP in urine (mg/h). The central estimates indicated in plots correspond to the posterior mode parameter set, the single best fitting parameter set over the 9 measures (3 outputs for each of 3 individuals) used for calibration. The shaded regions represent pointwise 95% credible intervals for the respective curves. This interval was derived by running each retained sample drawn from the posterior through the PBPK model and storing the output from each model output from 0 to 48 h in 0.05- hour increments. Output at each time point was retained and ordered with the 2.5th and 97.5th percentiles saved; the plotted 2.5% and 97.5% bounds are a smooth interpolation of these series of pointwise values.

**TABLE 6:** Comparisons of predictions and measured 48-h eliminations of 5OH-MEHTP, 2cx-MMHTP and 5cx-MEPTP for the three volunteers under the posterior mode parameter set.

	5OH-MEHTP		2cx-MMHTP		5cx-MEPTP	
	Measured	Predicted	Measured	Predicted	Measured	Predicted
V1	0.930	0.789	0.171	0.126	8.39	6.51
V2	0.527	0.622	0.110	0.126	4.76	5.54
V3	0.697	0.751	0.071	0.073	2.88	2.89

The estimates of 48-h metabolite excretions in urine (Table 6) were a close approximation to the measured volumes and the fit to BM data show the simulations were able to capture the distinct data trends in the three volunteers. However, the very high spikes at approximately 11- and 19-h following ingestion respectively for volunteer A could not be fitted by the present model form. High molecular weight plasticizers such as DINCH, DPHP and DEHTP are not readily absorbed from the gut and we speculate that these spikes were as a consequence of secondary uptake events from DEHTP residing in a lower section of the gastrointestinal tract, resulting from the consumption of food. Supplementary Figure S1 in supplementary material shows how small secondary uptake events simulated at 9.5- and 17-h following ingestion result in simulations that can approximate the BM data from this volunteer. However, there is no direct evidence to justify a modification to model form and formal calibration.

The traces corresponding to the posterior mode parameter set (Figures 4–6) either touching the bounds of the numerically derived confidence interval or even stepping outside at some points is likely a consequence of a) the pointwise method of calculating this interval and b) the significant variability in the shapes of curves resulting from the two uptake mechanisms. The deposition rates for the retained parameter sets that were within 1% of the posterior mode are illustrated for individual C in Supplementary Figure S2 of supplementary material and demonstrate significant variability in deposition rates post calibration: the ‘best estimate’ should not be over-interpreted—it is one of many that provide a similar quality of fit to data*.*

The lymphatic uptake and EHR are two relatively novel additions for PBPK models. Through first removing the lymphatic uptake and then also removing EHR, Supplementary Figures S3–S5 in supplementary materials show how accounting for these two processes was essential for fitting the complex trends seen in BM data.

### Parameter value uncertainty

Following calibration there was a substantial reduction in uncertainty (reduction in range of posterior distribution) relating to some global parameters, including the respective elimination rates of the three modelled second-order metabolites (K1_MOH, K1_2cx, K1_5cx), the half-lives of DEHTP in gut and liver (DEHTP_GUT_half_life and DEHTP_half_life) and two of the partition coefficients, Pbab and Pgub respectively. There was only a small reduction in uncertainty relating to other partition coefficients and to the individual-specific physiological parameters (Table 5). Since the subset of model parameters taken forward into calibration were determined by sensitivity analysis, the failure to substantially narrow parameter ranges, might appear surprising. However, in the prior specification the marginal parameter distributions were specified independently whereas parameters were all correlated in the posterior. In higher dimensional parameter space, there was in fact a very substantial narrowing of the posterior relative to the prior. In effect the inclusion of some of these parameters did not result in a better quality of fit to the available data, yet outputs were still sensitive to them and the failure to account for uncertainty in the full set of sensitive parameters would have resulted in a lower parameter value uncertainty in a reduced parameter subset than is justified.

A subset of parameters was more influential on concentration-time relationships for DEHTP and MEHTP in blood (principally the bound fraction of DEHTP and MEHTP (FB_DEHTP and FB_MEHTP respectively). Notably the upper ranges of FB_DEHTP and FB_MEHTP were reduced in the posterior distribution indicating that very high binding approaching 1 was inconsistent with urine data, however the marginal posterior distributions were otherwise relatively flat over the range 0.8–0.90. These results indicate the trends of DEHTP and MEHTP in blood are quite uncertain and would require specific data to allow for substantial refinement.

### Interpretation

Results from calibration suggest a minor fraction of DEHTP was absorbed—this was calculated for each individual as the sum of the hepatic and lymphatic fractions. Respective fractions (posterior median and 95% credible interval) of 0.363 (0.288, 0.468), 0.298 (0.261, 0.340) and 0.236 (0.206, 0.266) were calculated for individuals A-C, respectively. However, the PBPK model was coded to assume 100% of absorbed DEHTP was metabolised to MEHTP: if a significant fraction of DEHTP was metabolised directly to TPA, these fractions will be underestimates of the absorbed fractions of DEHTP. The significant uncertainty associated with these absorbed fractions reflects that there was no information on TPA concentrations in urine voids from the BM study data of (Lessmann et al., 2016), which is an non-specific yet a major downstream metabolite of DEHTP and thus the fractions of MEHTP metabolised to and eliminated as 5OH-MEHTP, 2cx-MMHTP and 5cx-MEPTP, were uncertain for each volunteer. Although the trends in deposition of these metabolites in urine could be captured, a significant range of absorbed fractions of DEHTP was consistent with the BM data following calibration.

The trends in metabolite deposition in urine (mg/hour) for the three volunteers each showed a double peak (Figures 4–6), (although for individual A this manifests as an inflection point), but otherwise these profiles were extremely variable, with large differences in deposition rates over time that reflect differences in the absorbed fractions and rates of uptake of DEHTP, and the respective fractions of DEHTP absorbed through hepatic and lymphatic routes. The PBPK model was able to successfully fit these distinct trends. The different uptake phases of DEHTP can be most easily observed in the data from individual B. The initial peak in rate of deposition occurs at approximately 3 h following ingestion for this individual (Figure 5) with the initial slow deposition rate corresponding to uptake of MEHTP from the first phase of the intestine and acceleration corresponding to uptake of DEHTP from the second phase of the intestine: these phases represent metabolism of DEHTP in gut and liver, respectively. The second peak at approximately 6 h following ingestion of DEHTP corresponds to uptake of DEHTP into the lymphatic system and the subsequent deposition into blood at the thoracic duct following a delay (representing transit through the lymphatic system). Whilst there was still a substantial uncertainty in the estimates of hepatic and lymphatic uptakes following calibration (0.031 (0.020, 0.072) and 0.263 (0.224, 0.296) respectively) results suggest that the lymphatic route was dominant for this volunteer. The results for individuals A and C were less clear-cut, however results (Table 5) suggested that hepatic uptake was greater than lymphatic uptake.

## Discussion

In this work we have presented the first available PBPK model for DEHTP. The structure of the model was based on the previously published PBPK model for DPHP (McNally et al., 2021; McNally and Loizou, 2023), with minor adaptions as necessary and initial model parameterisation based upon *in silico* and *in vitro* experimental data. The availability of a donor model for a similar chemical was significant since the model development and calibration for the DPHP model was based upon data from both timed blood measurements (concentrations of parent chemical and first metabolite) following ingestion of DPHP, and second order metabolites in urine. (DPHP has a mean Tanimoto similarity index of 0.704 with DEHTP, see Supplementary Materials for more details). The availability of two data streams enabled insights into the kinetics of DPHP that would not have been possible in the absence of data from either blood or urine voids. This understanding, with a few small adaptions to account for differences in the metabolic pathways of DEHTP relative to DPHP, was critical to model development. Global sensitivity analysis is recognised as an important tool in model development and testing (McNally et al., 2011; Loizou et al., 2015; Lumen et al., 2015). In this work a streamlined uncertainty and sensitivity analysis was possible since the donor model for DPHP had been extensively tested (McNally et al., 2021; McNally and Loizou, 2023).

Despite the extrapolation for model form and structure proving to be reasonable it is important to note the very large differences between the respective calibrated models. Whilst the inclusion of lymphatic uptake proved to be important for understanding the behaviour of DPHP in blood, this represented a very small fraction of the ingested chemical, approximately 1/8 of that which entered hepatic circulation; there was little evidence of this mechanism in data from urine voids (McNally and Loizou, 2023). In contrast, for DEHTP the fraction of ingested chemical entering lymphatic circulation was much greater and of a similar magnitude to that entering hepatic circulation; evidence for the dual uptake mechanisms was available from urine voids. In terms of the amounts absorbed by study participants, this was much higher for DEHTP relative to DPHP. Some caution is therefore necessary for this class of chemicals if attempting read across since basic adjustments to partition coefficients and metabolism parameters would be insufficient, even when the structure of the model itself is appropriate. On a related point we note that the *in silico* method (Lobell and Sivarajah, 2003) for predicting protein binding performed relatively poorly with an error in excess of two orders of magnitude (binding was predicted as in excess of 99.99%). Based on the PBPK model for DPHP where a similar magnitude of error was observed, it appears likely that this algorithm is systematically poor for highly lipophilic chemicals. As previously demonstrated with Hexamoll^®^ DINCH (diisononyl-cyclohexane-1, 2-dicarboxylate) (McNally et al., 2019) this has an important bearing on predictions of the persistence of parent chemical in venous blood—inferences on the behaviour of this class of highly lipophilic chemicals, based on calculations of chemical properties, should be made with extreme caution*.*

**FIGURE 7:**
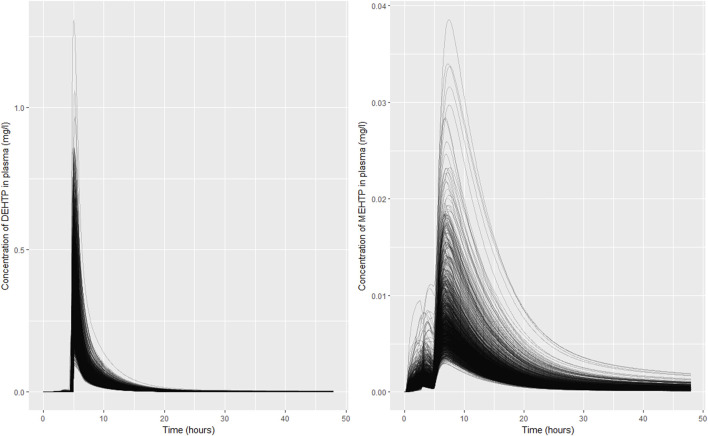
Simulated concentrations of **(A)** DEHTP in blood and **(B)** MEHTP in the blood of individual **(B)**. Individual curves correspond to the retained parameter sets from the posterior distribution.

Excellent fits to data from urine voids were achieved, indicating the model is credible, however there was significant uncertainty in the concentration time profiles of DEHTP and MEHTP, in blood. Whilst it was notable that the very high binding of DEHTP, predicted by log P, could be discounted, the peak concentration in blood and the elimination rate were still subject to significant uncertainty (Figure 7A). The shape profile for the concentration-time profile for MEHTP in blood (Figure 7B) was subject to greater uncertainty, however substantially lower blood concentrations were predicted for MEHTP relative to DEHTP. Concentration-response in venous blood data would be required to substantially reduce uncertainties in this aspect of the PBPK model, although some refinement of parameter ranges based upon expert judgement (for example by asserting that binding was lower for MEHTP compared to DEHTP) would yield some reduction in uncertainty. There is also potentially reducible uncertainty in tissue concentrations, particularly for MEHTP, however lower and upper bounds of key outputs such as peak concentrations and area-under-the-curve (AUC) could be estimated for comparison against *in vitro* toxicity data.

Lessmann et al. (2016) identified specific metabolites of DEHTP that could be used to infer population exposures to DEHTP based upon concentrations in spot urine voids (under certain assumptions). The total fractions of ingested DEHTP eliminated as these specific metabolites could be estimated from the study data and are sufficient for interpreting data from environmental monitoring. TPA could have been measured in the Lessmann et al. (2016) study, but as a non-specific metabolite of DEHTP, it was not analysed in urine voids. However, a PBPK model has more exacting requirements and, in particular, must account for 100% of the ingested substance. A simplified approach was developed to handle the technical problem arising from a network of metabolic pathways. TPA measurements would have been useful for the more exacting requirements of a PBPK model and could have yielded considerable reductions in uncertainty associated with absorbed fractions of DEHTP. Given that PBPK models are increasingly important for interpreting data from *in vitro* experiments (comparison of free, bioactive concentrations *in vitro* with *in vivo* tissue dosimetry e.g., in quantitative *in vitro* to *in vivo* (QIVIVE) studies (McNally and Loizou 2015; McNally et al., 2018; Loizou et al., 2021; Loizou et al., 2022)), a wider view of the potential usage of high-quality data from well conducted BM would be useful when designing controlled human BM studies. The BM studies for Hexamoll^®^ DINCH (Koch et al., 2013) and Di (2-ethylhexyl) adipate (DEHA) (Nehring et al., 2019; Nehring et al., 2020), where important non-specific metabolic products were measured in urine specimens, represent very good examples of study design for the specific requirements of PBPK modelling.

Finally, we note that the development of a human PBPK model with calibration using data from a human bio-monitoring study is the gold standard for evaluating ADME following chemical exposures, although we recognise this cannot be practically achieved for wide classes of chemicals. With this human model important insights into biological mechanisms could be inferred from simulations of a series of blood and urine specimen measurements. McNally et al. (2021) noted that a PBPK model for DPHP developed from blood specimens from the rat (Klein et al., 2016), with model form read across to humans, would have failed to capture important mechanisms. The human BM study reported in Klein et al. (2018) was critical for understanding the PK of this chemical. Based upon the similarity of PBPK model forms for DPHP and DEHTP, we confidently assert that a PBPK model developed from and calibrated to blood concentrations from animal experiments would likely have significant error in model form and would fail to identify the very significant inter-individual variation in the shape of the concentration-time relationship observed in a human population following single oral doses.

## Data Availability

The original contributions presented in the study are included in the article/Supplementary Material, further inquiries can be directed to the corresponding author.

## Footnotes

1
https://echa.europa.eu/registration-dossier/-/registered-dossier/15238/1/2.

2
https://www.tebu-bio.com/(26/01/2023).

3
https://www.acdlabs.com/.

4
https://www.gnu.org/software/mcsim/(as on 05/10/2022).

5
https://www.rstudio.com/products/rstudio/download/(as on 05/10/2022).

6
https://www.r-project.org/(as on 05/10/2022).

7
https://cran.r-project.org/web/packages/ggplot2/index.html (as on 05/10/2022).
