# Supplementary material for: Development, testing, parameterisation, and calibration of a human PBPK model for the plasticiser, di-(2-ethylhexyl) terephthalate (DEHTP) using in silico, in vitro and human biomonitoring data
Source: Front Pharmacol. 2023 Feb 20;14:1140852. doi: 10.3389/fphar.2023.1140852 (PMC9986446; doi:10.3389/fphar.2023.1140852)
Supplement: Supplementary file 1 [file DataSheet1.docx]

Supplementary material to ‘*Development, testing, parameterisation, and calibration of a human PBPK model for the plasticiser, Di-(2-ethylhexyl) terephthalate (DEHTP) using in silico, in vitro and human biomonitoring data’*

Kevin McNally^a^, Craig Sams^a^, Alex Hogg^a^, and George Loizou^a^

Authors’ affiliation

^a^ Health and Safety Executive, Harpur Hill, Buxton, UK

### Mass Spectrometry

| Table S1. MS parameters |  |
| --- | --- |
| Ion spray voltage | -4500v |
| Curtain gas | 35 |
| Temperature | 450^O^C |
| Declustering potential (DP) | -50v |
| Entrance potential (EP) | -6v |
| Collision energy (CE) | -22v |

**Table S2. Tanimoto Similarities^[[1]](#footnote-1)^**

|  |  | Similarity | | | | | |
| --- | --- | --- | --- | --- | --- | --- | --- |
| Plasticiser |  | morgan | fmorgan | maccs | atompairs | avalon | mean |
| Di(2-ethylhexyl) terephthalate  (DEHTP) | 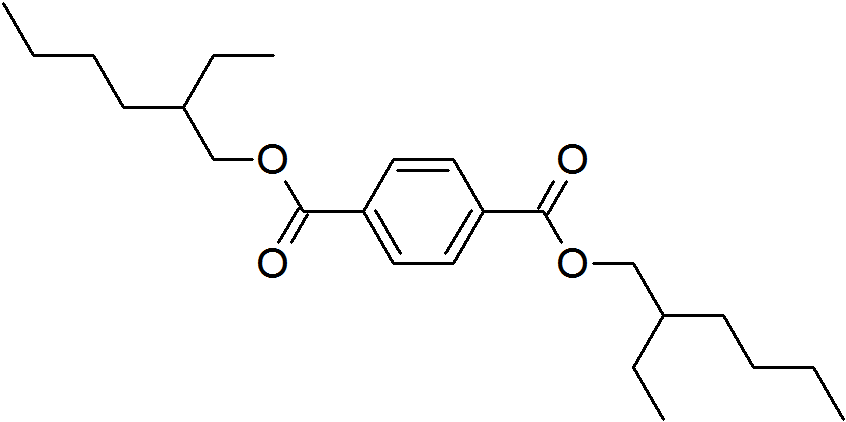 | 1 | 1 | 1 | 1 | 1 | 1 |
| Di(2-propylheptyl) phthalate  (DPHP) | 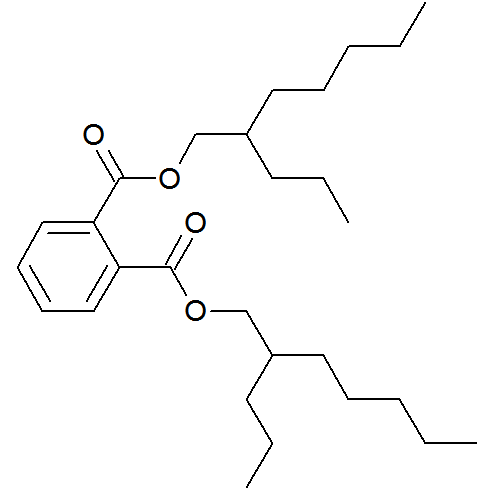 | 0.536 | 0.833 | 0.931 | 0.553 | 0.667 | 0.704 |
| (DEHP) Di(2-Ethylhexyl) phthalate  (DEHP) | 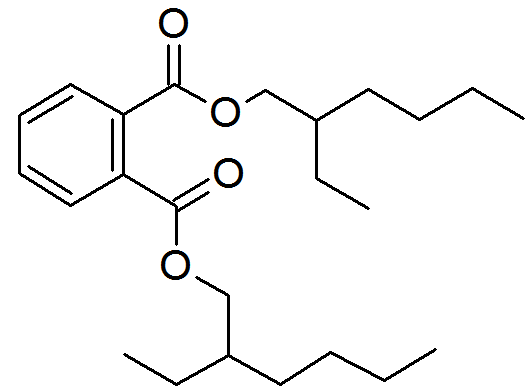 | 0.7222 | 0.875 | 0.931 | 0.632 | 0.807 | 0.794 |

### Supplementary analysis

Figure S1 shows results from supplementary analysis on volunteer 1 that aimed to investigate whether secondary uptake events of DEHTP from lower sections of the GI tract could potentially explain two very large peaks in this volunteer’s BM data at approximately 11 and 19 hours following ingestion of DEHTP. The presentation of results follows that in Figure 4 - Figure 6; the central estimate and numerically derived confidence interval shown in Figure S1 for rates of deposition of OH-MEHTP, 2cx-MMHTP and 5cx-MEPTP into urine (mg/h) were generated using the same numerical procedure described in the main text of results. The results in Figure S1 are based on the exact same parameter sets used to produce Figure 4, however the model was modified to code uptake events at 9.7 and 17 hours following ingestion of DEHTP. An improved fit to the entire dataset could be likely be achieved through re-running calibration with this modified model, however given that this innovation is somewhat speculative it would be difficult to justify this model form.

Figure S2 shows rates of deposition of OH-MEHTP, 2cx-MMHTP and 5cx-MEPTP in urine for volunteer 3 for the 29 parameter sets that were within 1% of the posterior mode. These traces show significant variabilities in deposition profiles that were consistent with the BM data.

Figures S3 – S5 show rates of deposition of OH-MEHTP, 2cx-MMHTP and 5cx-MEPTP into urine (mg/h) corresponding to volunteer 3. In each figure the top left panel corresponds to results for these metabolites shown in Figure 6 of the manuscript. The top right panel of each figure shows the effect of removing the lymphatic uptake component – samples from the posterior were used to generate this plot, but with the lymphatic fraction of dose overwritten by zero in each simulation. The bottom left panel of each figure shows the additional effort of removing EHR: the lymphatic fraction of dose and the uptake into bile were both overwritten by zero when generating these simulations. From these results the effects of modelling uptake into the lymphatic system and of modelling EHR can be clearly appreciated.


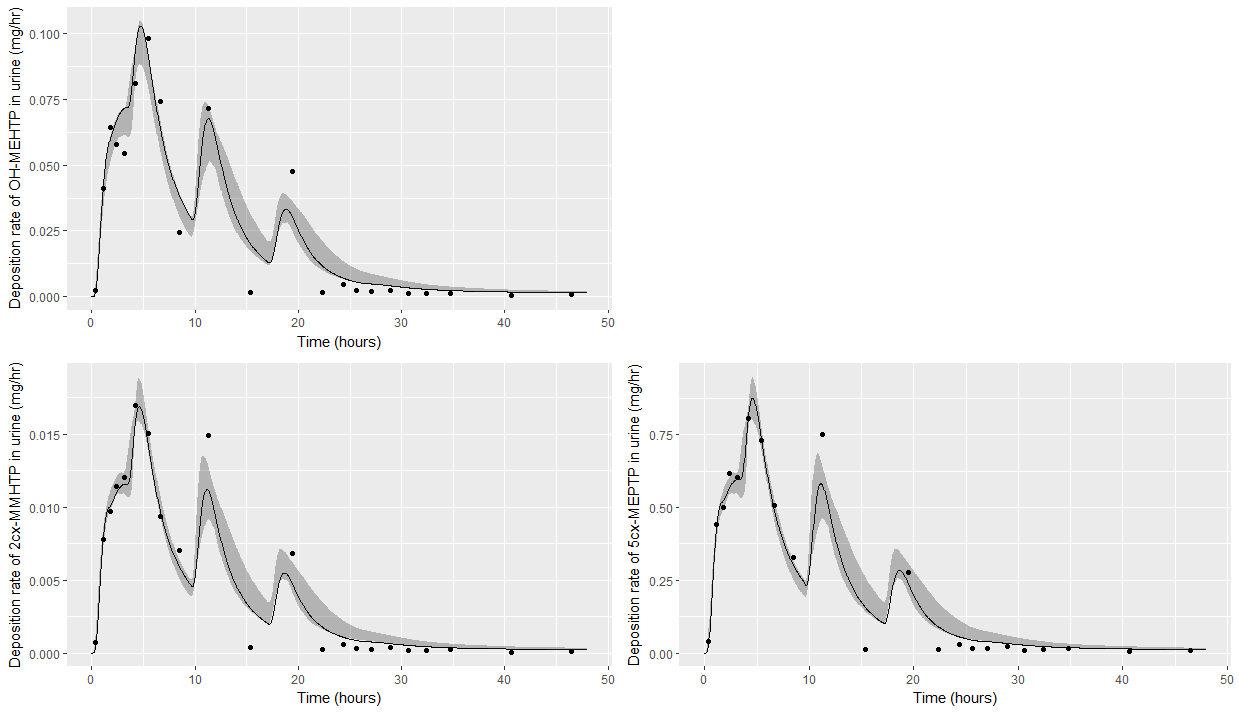


Figure S1: Simulated rates of deposition into urine of OH-MEHTP, 2cx-MMHTP and 5cx-MEPTP (mg/h) for volunteer 1 with two secondary uptake events from the gut coded at 9.5 and 17 hours following ingestion of DEHTP.


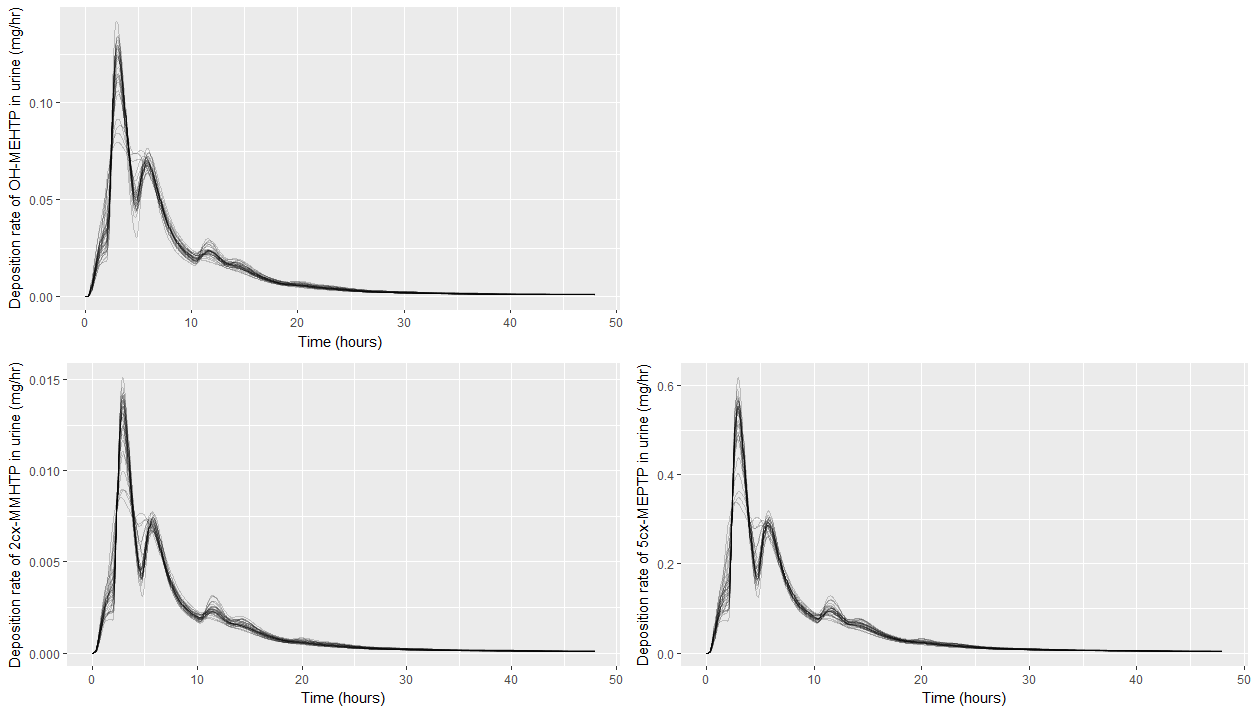


Figure S2: Simulated rates of deposition into urine of OH-MEHTP, 2cx-MMHTP and 5cx-MEPTP (mg/h) for volunteer 3 corresponding to the 29 parameter sets that were within 1% of the posterior mode


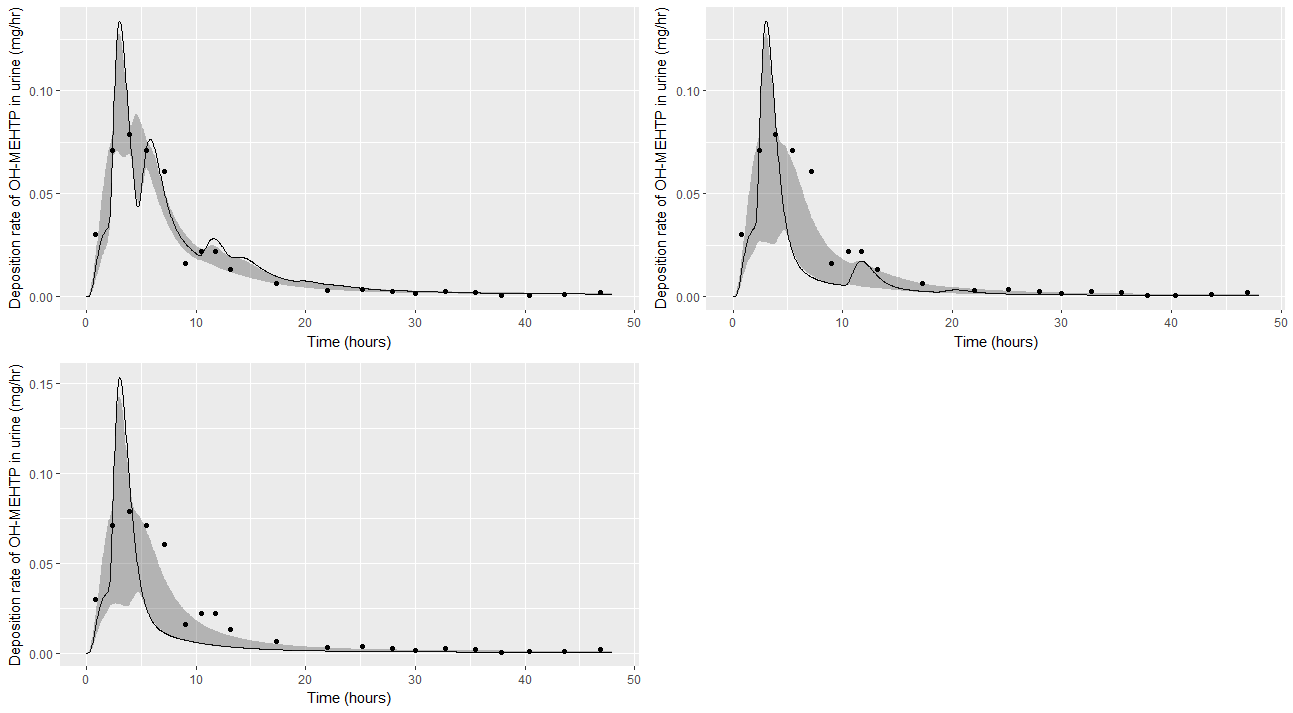


Figure S3: Simulated rates of deposition into urine of OH-MEHTP (mg/h) for volunteer 3. The panels correspond to: calibrated model (top left); calibrated model with lymphatic uptake set to zero (top right); calibrated model with lymphatic uptake set to zero and EHR set to zero (bottom left)


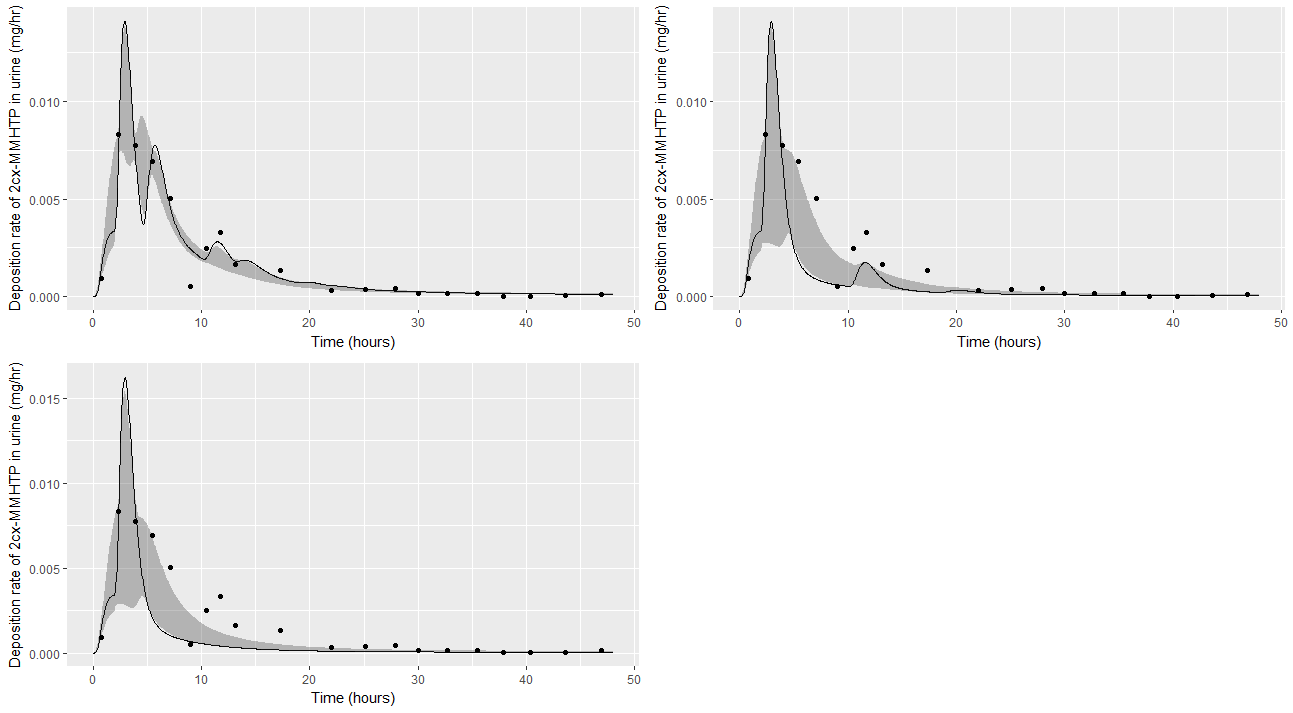


Figure S4: Simulated rates of deposition into urine of 2cx-MMHTP (mg/h) for volunteer 3. The panels correspond to: calibrated model (top left); calibrated model with lymphatic uptake set to zero (top right); calibrated model with lymphatic uptake set to zero and EHR set to zero (bottom left)


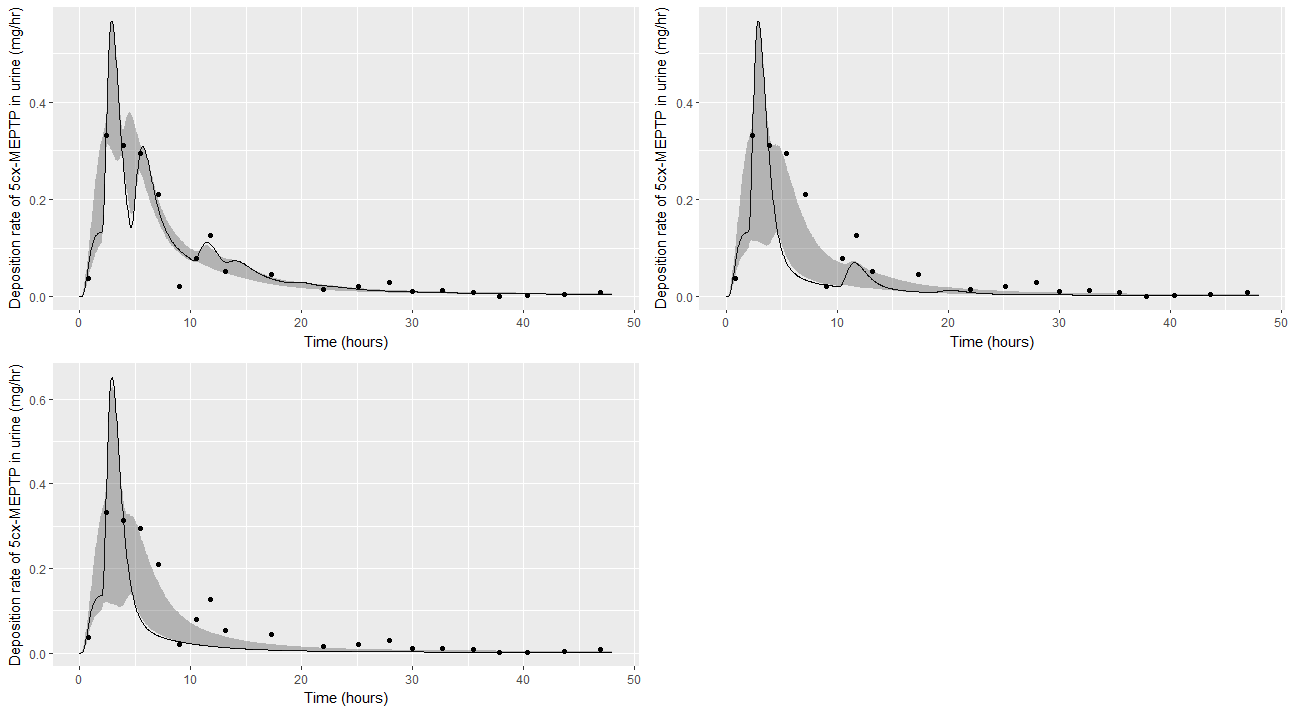


Figure S5: Simulated rates of deposition into urine of 5cx-MEPTP (mg/h) for volunteer 3. The panels correspond to: calibrated model (top left); calibrated model with lymphatic uptake set to zero (top right); calibrated model with lymphatic uptake set to zero and EHR set to zero (bottom left)

## MCSim code for DEHTP

# MCSim script

# Di(2-ethylhexyl) terephthalate (DEHTP)

# George Loizou and Kevin McNally (HSE)

# Compiled on: 01/11/2022

States =

{

Afa,

Agu1,

Agu2,

AMgu,

Ast,

Arpd,

Aspd,

Ali,

Aki,

AMli,

ABile,

Alymph,

ARBC_DEHTP,

Aplasm_DEHTP,

ABellyH,

AGiTractH,

ABowel,

ABellylymph,

AGiTractlymph,

AfaM,

AstM,

AguM,

AMliM,

AliM,

AkiM,

ABileM,

ABowelM,

AspdM,

ArpdM,

Aplasm_MEHTP,

ARBC_MEHTP,

AMMEHTPB_MOH,

AMMEHTPB_2cx,

AMMEHTPB_5cx,

AMMEHTPU_MOH,

AMMEHTPU_2cx,

AMMEHTPU_5cx,

VBladder,

Gutswitch,

Lymphswitch,

DOSESTEP

};

Outputs =

{

Cli,

Cki,

Cfa,

Cgu1,

Cgu2,

Cst,

Clymph,

Cspd,

Crpd,

mass,

Uptake,

reloral,

CVfa,

CVgu1,

CVgu2,

CVst,

CVspd,

CVli,

CVki,

CVrpd,

CV,

CVnmol,

CA_DEHTP,

CAT_DEHTP,

CRBC_DEHTP,

CV_total_nmol,

CVM,

CliM,

CkiM,

CVliM,

CVkiM,

CguM,

CVguM,

CstM,

CVstM,

CfaM,

CVfaM,

CspdM,

CVspdM,

CrpdM,

CVrpdM,

CA_MEHTP,

CAT_MEHTP,

CRBC_MEHTP,

CV_total_MEHTP_nmol,

relMEHTP,

massMEHTP,

Curine_MEHTP,

Curine_MOH,

Curine_2cx,

Curine_5cx,

ODOSEliver,

ODOSElymph,

ODOSEbowel,

ClintDEHTP,

ClintDEHTPgu,

ClintMEHTP,

Ali_lag,

AliM_lag,

AMguOut,

AMliOut,

Blood_DEHTP,

Blood_MEHTP,

Urine_2cx,

Urine_5cx,

Urine_OH,

Urine_MEHTP

};

Inputs =

{

events_Gutswitch,

events_Lymphswitch,

events_DOSESTEP,

events_VBladder,

events_AMMEHTPU_MOH,

events_AMMEHTPU_2cx,

events_AMMEHTPU_5cx

};

# Parameters

BW = 89; # body weight (kg)

MWDEHTP = 390.56; # DEHTP molecular mass (g/mol)

MWMEHTP = 101.97; # MEHTP molecular mass (g/mol)

MWMEHTP5OH = 294.34; # MEHTP5OH molecular mass (g/mol)

MWMEHTP2cx = 308.33; # 2cx-MEHTP molecular mass (g/mol)

MWMEHTP5cx = 308.33; # 5cx-MEHTP molecular mass (g/mol)

CAE = 0.75; # cardiac allometric exponent

QCC = 11.22; # cardiac allometric constant (L/h/kg^CAE)

VT = 0.95; # proportion of vascularised tissue

VfaC = 0.195; # fractional volume fat

VguC = 0.067; # fractional volume gut

VstC = 0.0158; # fractional volume stomach

VspdC = 0.4714; # fractional volume poorly perfused

VrpdC = 0.0305; # fractional volume richly perfused

VliC = 0.0203; # fractional volume liver

VkiC = 0.0025; # fractional volume kidney

VlymphC = 0.0036; # lymph system fractional volume

VBldC = 0.05; # blood fractional volume

QhepartC = 0.06; # hepatic artery fractional blood flow

QguC = 0.17; # fractional blood flow gut

QstC = 0.01; # fractional blood flow stomach

QspdC = 0.27; # overall fractional blood flow to slowly perfused tissue

QrpdC = 0.22; # overall fractional blood flow to rapidly perfused tissue

QkiC = 0.2; # fractional blood flow to kidney

QfaC = 0.05; # fractional blood flow to fat

FracDOSELymph = 0.05; # Fraction of dose taken into lymph

FracDOSEHep = 0.1; # Fraction of dose taken into hepatic

FracMetabMOH = 0.024; # Fraction of CYP-mediated metabolism MEHTP -> MEHTP5OH

FracMetab2cx = 0.004; # Fraction of CYP-mediated metabolism MEHTP -> MWMEHTP2cx

FracMetab5cx = 0.204; # Fraction of CYP-mediated metabolism MEHTP -> MWMEHTP5cx

FB_DEHTP = 0.99984; # Fraction of DEHTP bound to plasma proteins

FB_MEHTP = 0.9854; # Fraction of MEHTP bound to plasma proteins

PORALDOSE = 0.59; # oral dose [mg/kg]

DRINKTIME = 0.25; # Drink time [h]

BELLYPERM = 0.685; # [/h]

GIPERM1 = 5.1; # [/h]

GIPERM2 = 5.1; # [/h]

BELLYPERMlymph = 0.685; # [/h]

GIPERMlymph = 5.1; # [/h]

KEMAX = 10.2; # [Maximum emptying rate /h]

KEMIN = 0.005; # [Minimum emptying rate /h]

KA_MEHTP = 0.3; # 1st-order oral uptake rate of MEHTP (1/hr)

Lymphswitch = 1;

Gutswitch = 1;

MPY = 34; # microsomal protein yield [mg microsomal protein/g liver]

MPYgu = 3.9; # microsomal protein yield [mg microsomal protein/g gut]

Incub_vol = 1; # Volume of incubation (ml)

Microsome_prot = 0.5; # microsomal protein amount (mg)

DEHTP_half_life = 3; # DEHTP -> MEHTP half-life (minutes)

DEHTP_GUT_half_life = 60; # DEHTP -> MEHTP GUT half-life (minutes)

MEHTP_half_life = 66.316; # MEHTP -> OH-MEHTP and cx-MEHTP half-life (minutes)

RUrine = 0.1; # Rate of Urine Production [l/h]

Creat = 1.217; # Urinary creatinine concentration [g/L] or 0.01192 [mol/L]

K1_MOH = 0.1; # First-order elimination rate from blood [/h]

K1_2cx = 0.1; # First-order elimination rate from blood [/h]

K1_5cx = 0.1; # First-order elimination rate from blood [/h]

K1_MEHTP = 0.0; # First-order elimination rate from blood [/h]

K1_DEHTP_GUT = 0; # First-order elimination rate of DEHTP from gut into bowel [/h]

K1_DEHTP_LIVER = 10; # First-order elimination rate of MEHTP from liver into bile [/h]

K1_MEHTP_GUT = 0; # First-order elimination rate of MEHTP from gut into bowel [/h]

K1_MEHTP_LIVER = 0; # First-order elimination rate of MEHTP from liver into bile [/h]

K1Lymph = 0.2; # First-order elimination rate from Lymph into blood [/h]

Lymphlag = 3.01; # Lag between uptake into Lymph and emptying into blood [h]

Gutlag = 3.01; # Lag between uptake into GItract and emptying into gut [h]

Pbab = 3.01; # DEHTP Blood air partition coefficient

Pfab = 63.38; # DEHTP Fat tissue:air partition coefficient

Pgub = 7.4; # DEHTP GI Tract tissue:blood partition coefficient

Pstb = 7.4; # DEHTP Stomach tissue:air partition coefficient

Prpdb = 3.7; # DEHTP Richly tissue:air partition coefficient

Pkib = 3.7; # DEHTP Kidney tissue:blood partition coefficient

Pspdb = 3.29; # DEHTP Slowly perfused tissue:air partition coefficient

Plib = 5.89; # DEHTP Liver tissue:air partition coefficient

Prbcb = 30; # DEHTP Red blood cells:plasma partition coefficient

PbaM = 3.01; # MEHTP blood:air partition coefficient

PspdM = 3.29; # MEHTP Slowly perfused tissue:air partition coefficient

PliM = 5.89; # MEHTP Liver tissue:air partition coefficient

PrpdM = 3.69; # MEHTP Richly tissue:air partition coefficient

PkiM = 12.20; # MEHTP Kidney tissue:blood partition coefficient

PfaM = 20.32; # MEHTP Fat tissue:air partition coefficient

PstM = 7.39; # MEHTP Stomach tissue:air partition coefficient

PguM = 7.39; # MEHTP GI Tract tissue:blood partition coefficient

PrbcM = 30; # MEHTP Red blood cells:plasma partition coefficient

Vfa = 0;

Vgu = 0;

Vst = 0;

Vspd = 0;

Vrpd = 0;

Vli = 0;

Vki = 0;

Vlymph = 0;

Qfa = 0;

Qgu = 0;

Qst = 0;

Qrpd = 0;

Qspd = 0;

Qki = 0;

Qli = 0;

QCMC = 0;

ODOSEliver = 0;

ODOSElymph = 0;

ODOSEbowel = 0;

Uptake = 0;

Vplas = 0;

VRB = 0;

Vbld = 0;

Qhepart = 0;

CA_DEHTP = 0;

CAT_DEHTP = 0;

CA_CRBC = 0;

CVnmol = 0;

CV_total_nmol = 0;

CVM = 0;

CA_MEHTP = 0;

CAT_MEHTP = 0;

#CRBC_MEHTP = 0;

CV_total_MEHTP_nmol = 0;

escapeFrac = 0.05;

escapeFrac2 = 0.05;

# SD terms for MCMC

Sigma1 = 0.1;

Sigma2 = 0.1;

Sigma3 = 0.1;

Initialize

{

BWc = pow(BW, CAE); # cardiac scaling output factor (kg)

VplasC = 0.55 * VBldC; # plasma fractional volume

HEME = 1 - (VplasC / VBldC); # Volume of Haeme

VRBC = HEME * VBldC; # Volume of red blood cells

## Gelman reparameterisations

Qcci = QrpdC + QspdC + QhepartC + QfaC + QstC + QguC + QkiC;

Qrpdci = QrpdC / Qcci;

Qspdci = QspdC / Qcci;

Qhepartci = QhepartC / Qcci;

Qkici = QkiC/Qcci;

Qfaci = QfaC / Qcci;

Qstci = QstC / Qcci;

Qguci = QguC / Qcci;

Vti = (1 - VT) + VrpdC + VspdC + VliC + VfaC + VstC + VguC + VplasC + VRBC + VlymphC + VkiC;

Vguci = VguC / Vti;

Vstci = VstC / Vti;

Vfaci = VfaC / Vti;

Vlici = VliC / Vti;

Vkici = VkiC / Vti;

Vspdci = VspdC / Vti;

Vrpdci = VrpdC / Vti;

Vbldci = VBldC / Vti;

Vplasci = VplasC / Vti;

VRBCci = VRBC / Vti;

Vlymphci = VlymphC / Vti;

# Volumes scaled to actual volumes

Vfa = Vfaci * BW; # scaled fractional volume

Vgu = Vguci * BW; # scaled fractional volume

Vst = Vstci * BW; # scaled fractional volume

Vspd = Vspdci * BW; # scaled fractional volume

Vli = Vlici * BW; # scaled fractional volume

Vki = Vkici * BW; # scaled fractional volume

Vrpd = Vrpdci * BW; # scaled fractional volume

Vlymph = Vlymphci * BW; # scaled fractional volume

VRB = VRBCci * BW; # scaled red blood cell fractional volume

Vplas = Vplasci * BW; # plasma fractional volume

Vbld = Vbldci * BW; # Whole blood fractional volume

# Calculate actual blood flows from total flow and percent flows

QC = QCC * BWc; # cardiac output (L/h)

Qfa = Qfaci * QC; # scaled fractional blood flow

Qgu = Qguci * QC; # scaled fractional blood flow

Qst = Qstci * QC; # scaled fractional blood flow

Qrpd = Qrpdci * QC; # scaled fractional blood flow

Qki = Qkici * QC; # scaled fractional blood flow

Qspd = Qspdci * QC; # scaled fractional blood flow

Qhepart = Qhepartci * QC; # scaled hepatic artery fractional blood flow

Qli = Qhepart + Qst + Qgu; # scaled fractional blood flow

QCMC = Qhepart + Qgu + Qst + Qfa + Qrpd + Qspd + Qki;

} # End of model initialization

Dynamics

{

tau = 8; # the required delay

Ali_lag = CalcDelay(Ali, tau);

AliM_lag = CalcDelay(AliM, tau);

ORALDOSE = PORALDOSE * BW; # scaled oral dose (mg/day)

DOSEFLOW = ORALDOSE / DRINKTIME; # zero order uptake rate constant

ODOSE = DOSEFLOW * DOSESTEP; # amount absorbed (mg)

ODOSEliver = ODOSE * FracDOSEHep;

ODOSElymph = ODOSE * FracDOSELymph;

ODOSEbowel = ODOSE * (1 - FracDOSEHep - FracDOSELymph);

ClintDEHTP = (0.693 / DEHTP_half_life) * (Incub_vol / Microsome_prot) * MPY * Vli * 60; # Clearance (L/h whole liver)

ClintDEHTPgu = (0.693 / DEHTP_GUT_half_life) * (Incub_vol / Microsome_prot) * MPYgu * Vgu * 60; # Clearance (L/h gut)

ClintMEHTP = (0.693 / MEHTP_half_life) * (Incub_vol / Microsome_prot) * MPY * Vli * 60; # Clearance (L/h whole liver)

#DEHTP Concentrations in Compartments

# cellular concentrations (mg/L)

Cfa = Afa / Vfa;

Cgu1 = Agu1 / Vgu;

Cgu2 = Agu2 / Vgu;

Cst = Ast / Vst;

Cspd = Aspd / Vspd;

Crpd = Arpd / Vrpd;

Cli = Ali / Vli;

Cki = Aki / Vki;

Clymph = Alymph / Vlymph;

# venous organ concentration (mg/L)

CVfa = Cfa / Pfab;

CVgu1 = Cgu1 / Pgub;

CVgu2 = Cgu2 / Pgub;

CVst = Cst / Pstb;

CVspd = Cspd / Pspdb;

CVli = Cli / Plib;

CVki = Cki / Pkib;

CVrpd = Crpd / Prpdb;

GPER = KEMAX / (1 + KEMIN * Cst);

# venous concentration (mg/L)

CV = ((CVfa * Qfa) + (CVrpd * Qrpd) + (CVspd * Qspd) + (CVki * Qki) + (CVli * Qli)) / QCMC;

# DEHTP Venous concentration (nmoles/L)

CVnmol = (CV / MWDEHTP) * 1000000;

# Fraction unbound

Aplasmub_DEHTP = Aplasm_DEHTP * (1 - FB_DEHTP);

# mass in system (kg)

mass =

ARBC_DEHTP + Aplasm_DEHTP + AMli + Ali + ABile + AMgu + ABellyH + AGiTractH +

Ast + Agu1 + Agu2 + ABowel + ABellylymph + AGiTractlymph + Alymph + Afa + Arpd +

Aspd + Aki;

Uptake = ODOSEliver + ODOSElymph + ODOSEbowel;

# mass balance

reloral = ((t>0) ? mass / (ORALDOSE + 1e-10) : 1);

CA_DEHTP = Aplasmub_DEHTP / (Vplas); # Arterial unbound concentration (nmol/L)

CRBC_DEHTP = ARBC_DEHTP / VRB;

#MEHTP Concentrations in Compartments

# cellular concentrations (mg/L)

CguM = AguM / Vgu;

CstM = AstM / Vst;

CfaM = AfaM / Vfa;

CliM = AliM / Vli;

CkiM = AkiM / Vki;

CspdM = AspdM / Vspd;

CrpdM = ArpdM / Vrpd;

# venous organ concentration (mg/L)

CVguM = CguM / PguM;

CVstM = CstM / PstM;

CVfaM = CfaM / PfaM;

CVliM = CliM / PliM;

CVkiM = CkiM / PkiM;

CVspdM = CspdM / PspdM;

CVrpdM = CrpdM / PrpdM;

CVM = ((CVliM * Qli) + (CVfaM * Qfa) + (CVspdM * Qspd) + (CVkiM * Qki) + (CVrpdM * Qrpd)) / QCMC;

#unbound model

Aplasmub_MEHTP = Aplasm_MEHTP * (1 - FB_MEHTP);

CA_MEHTP = Aplasmub_MEHTP / (Vplas);

CRBC_MEHTP = ARBC_MEHTP / VRB;

# mass in system (kg)

massMEHTP = AguM + AstM + AfaM + AMliM + AspdM + ArpdM + ABowelM + ABileM + AkiM + ARBC_MEHTP + Aplasm_MEHTP;

# MEHTP mass balance

relMEHTP = ((t>0) ? massMEHTP / (AMli + AMgu + 1e-10) : 1);

#DEHTP Differential Equations

dt (Gutswitch) = 0;

dt (Lymphswitch) = 0;

dt (DOSESTEP) = 0;

dt (VBladder) = RUrine;

dt (ARBC_DEHTP) = (CA_DEHTP - CRBC_DEHTP / Pbab); # Amount in red blood cells

dt (Aplasm_DEHTP) = QCMC * (CV - CA_DEHTP) - dt (ARBC_DEHTP) + Lymphswitch * Alymph * K1Lymph; # Amount in plasma

dt (AMli) = ((Qli * ClintDEHTP) / (Qli + ClintDEHTP / Pbab)) * CVli; # Amount of hepatic metabolism

dt (Ali) = (Qhepart * CA_DEHTP) + (Qst * CVst) + (Qgu * CVgu2) - (Qli * CVli) - dt (AMli) -

(K1_DEHTP_LIVER * Ali); # Amount in liver

dt (ABile) = K1_DEHTP_LIVER * (Ali - Ali_lag); # Amount in Bile

dt (AMgu) = ((Qgu * ClintDEHTPgu) / (Qgu + ClintDEHTPgu / Pbab)) * CVgu1; # Amount of gut metabolism

dt (ABellyH) = (ODOSEliver) - (GPER * ABellyH) - (BELLYPERM * ABellyH); # Amount in belly (hepatic)

dt (AGiTractH) = (GPER * ABellyH) - (GIPERM1 * AGiTractH); # Amount in GI Tract compartment (hepatic)

dt (Ast) = Qst * (CA_DEHTP - CVst) + BELLYPERM * ABellyH; # Amount in stomach compartment

dt (Agu1) = (GIPERM1 * AGiTractH) - dt (AMgu) - (Gutswitch * GIPERM2 * Agu1) ; # Amount in first gut compartment

dt (Agu2) = Qgu * (CA_DEHTP - CVgu2) + (Gutswitch * GIPERM2 * Agu1) - (Lymphswitch * K1_DEHTP_GUT * Agu2) + (K1_DEHTP_LIVER * Ali_lag); # Amount in second gut compartment

dt (ABowel) = ODOSEbowel + (Lymphswitch * K1_DEHTP_GUT * Agu2); # Elimination from gut into faeces

dt (ABellylymph) = (ODOSElymph) - (GPER * ABellylymph) - (BELLYPERMlymph * ABellylymph); # # Amount in (lymph)

dt (AGiTractlymph) = (GPER * ABellylymph) - (GIPERMlymph * AGiTractlymph); # DEHTP rate of uptake in lymph compartment (mg/h/kg) # Amount in GI Tract (lymph)

dt (Alymph) = (BELLYPERMlymph * ABellylymph) + (GIPERMlymph * AGiTractlymph) -

Lymphswitch * Alymph * K1Lymph; # Amount in lymph

dt (Afa) = Qfa * (CA_DEHTP - CVfa); # Amount in fat

dt (Arpd) = Qrpd * (CA_DEHTP - CVrpd); # Amount in RPD

dt (Aspd) = Qspd * (CA_DEHTP - CVspd); # Amount in SPD

dt (Aki) = Qki * (CA_DEHTP - CVki); # Amount in kidney

# MEHTP Differential Equations

dt (AMliM) = ((Qli * ClintMEHTP) / (Qli + ClintMEHTP / PbaM)) * CVliM; # Amount of hepatic metabolism

dt (AliM) = (Qhepart * CA_MEHTP) + (Qst * CVstM) + (Qgu * CVguM) - (Qli * CVliM) +

((1 - escapeFrac2) * dt (AMli)) - dt (AMliM) - (K1_MEHTP_LIVER * AliM); #Amount in liver

dt (ABileM) = K1_MEHTP_LIVER * (AliM - AliM_lag); #Amount in bile

dt (AstM) = Qst * (CA_MEHTP - CVstM); #Amount in stomach

dt (AguM) = Qgu * (CA_MEHTP - CVguM) + ((1 - escapeFrac) * dt (AMgu)) - (Lymphswitch * K1_MEHTP_GUT * AguM) + (K1_MEHTP_LIVER * AliM_lag); #Amount in gut

dt (ABowelM) = K1_MEHTP_GUT * AguM; # Elimination from gut into faeces

dt (AfaM) = Qfa * (CA_MEHTP - CVfaM); # Amount in fat

dt (ArpdM) = Qrpd * (CA_MEHTP - CVrpdM); # Amount in RPD

dt (AspdM) = Qspd * (CA_MEHTP - CVspdM); # Amount in SPD

dt (AkiM) = Qki * (CA_MEHTP - CVkiM) - K1_MEHTP*AkiM; #Amount in kidney

dt (ARBC_MEHTP) = (CA_MEHTP - CRBC_MEHTP / PbaM); # Amount in red blood cells

dt (Aplasm_MEHTP) = QCMC * (CVM - CA_MEHTP) - dt (ARBC_MEHTP) + (escapeFrac * dt (AMgu)) + (escapeFrac2 * dt (AMli)); # Amount in plasma

#Urinary excretion of metabolites

dt (AMMEHTPB_MOH) =

dt (AMliM) * FracMetabMOH * (MWMEHTP / MWDEHTP) * (MWMEHTP5OH / MWMEHTP) -

(K1_MOH * AMMEHTPB_MOH); # OH in blood

dt (AMMEHTPB_2cx) =

dt (AMliM) * FracMetab2cx * (MWMEHTP / MWDEHTP) * (MWMEHTP2cx / MWMEHTP) -

(K1_2cx * AMMEHTPB_2cx); # 2cx in blood

dt (AMMEHTPB_5cx) =

dt (AMliM) * FracMetab5cx * (MWMEHTP / MWDEHTP) * (MWMEHTP5cx / MWMEHTP) -

(K1_5cx * AMMEHTPB_5cx); # 5cx in blood

dt (AMMEHTPU_MOH) = K1_MOH * AMMEHTPB_MOH; #OH in urine

dt (AMMEHTPU_2cx) = K1_2cx * AMMEHTPB_2cx; # 2cx in urine

dt (AMMEHTPU_5cx) = K1_5cx * AMMEHTPB_5cx; # 5cx in urine

Curine_MOH = K1_MOH * AMMEHTPB_MOH;

Curine_2cx = K1_2cx * AMMEHTPB_2cx;

Curine_5cx = K1_5cx * AMMEHTPB_5cx;

Curine_MEHTP = K1_MEHTP*AkiM* (MWMEHTP / MWDEHTP);

}

CalcOutputs

{

CAT_DEHTP = Aplasm_DEHTP/(MWDEHTP*Vplas)*1e6; # Total concentration DEHTP in plasma (nmol/l)

CV_total_nmol = (Aplasm_DEHTP+ARBC_DEHTP)/(MWDEHTP*Vbld)*1e6; # Total concentration DEHTP in blood (nmol/l)

CRBC_DEHTP = ARBC_DEHTP / (MWDEHTP*VRB)*1e6; # Concentration in red blood cells (nmol/L)

CAT_MEHTP = Aplasm_MEHTP / (MWMEHTP*Vplas)*1e6 * (MWMEHTP / MWDEHTP); # Total concentration MEHTP in plasma (nmol/l)

CRBC_MEHTP = ARBC_MEHTP / (MWMEHTP*VRB)*1e6 * (MWMEHTP / MWDEHTP); # Total concentration MEHTP in red blood cells (nmol/l)

CV_total_MEHTP_nmol = (Aplasm_MEHTP+ARBC_MEHTP)/(MWMEHTP*Vbld)*1e6 * (MWMEHTP / MWDEHTP); # Total concentration MEHTP in blood (nmol/l)

Blood_DEHTP = (Aplasm_DEHTP)/(Vbld); #Total concentration of DEHTP in blood (mg/l)

Blood_MEHTP = (Aplasm_MEHTP)/(Vbld) * (MWMEHTP / MWDEHTP); #Total concentration of MEHTP in blood (mg/l)

Blood_2cx = AMMEHTPB_2cx/(Vbld); # Total concentration of 2cx in blood (mg/l)

Blood_5cx = AMMEHTPB_5cx/(Vbld); # Total concentration of 5cx in blood (mg/l)

Blood_OH = AMMEHTPB_MOH/(Vbld); # Total concentration of OH in blood (mg/l)

Urine_2cx = AMMEHTPB_2cx*K1_2cx; #Deposition rate of 2cx into urine

Urine_5cx = AMMEHTPB_5cx*K1_5cx; #Deposition rate of 5cx into urine

Urine_OH = AMMEHTPB_MOH*K1_MOH; #Deposition rate of OH into urine

Urine_MEHTP = K1_MEHTP*AkiM * (MWMEHTP / MWDEHTP); #Rate of deposition of MPHP in urine (mg/h)

}

End.

1. The Tanimoto similarities were obtained by calculating a mean of five different fingerprints (Morgan, FeatMorgan, MACCS, Atom Pair, and Avalon). Molecular fingerprinting was obtained using RDKit nodes implemented in KNIME v. 4.6.4. [↑](#footnote-ref-1)
